# Reversible phase transition in 8,19-dimethyl-2,3,8,19-tetra­aza­penta­cyclo[13.7.0.0^4,12^.0^6,10^.0^17,21^]docosa-1(15),2,4(12),5,10,16,21-hepta­ene-7,9,18,20-tetrone

**DOI:** 10.1107/S2056989025003998

**Published:** 2025-05-09

**Authors:** Christian Näther, Artjom Businski, Rainer Herges

**Affiliations:** a Institut für Anorganische Chemie, Universität Kiel, Germany; b Otto-Diels-Institut für Organische Chemie, Universität Kiel, Germany; University of Aberdeen, United Kingdom

**Keywords:** diazo­cine, photoswitch, phase transition, reversibility, temperature-dependent measurements, twinning, crystal structure

## Abstract

The crystal structure of the title compound, C_20_H_14_N_4_O_4_, was previously reported at 220 K [Businski *et al.* (2025 ▸). *Chem. Eur. J.* A**71**, 3–8], where it crystallizes in the monoclinic space group *P*2_1_/*c* with one crystallographically independent mol­ecule in a general position. In this structure, disorder of the methyl H atoms is observed. However, upon cooling a continuous splitting of the reflections occurs, that might indicate a phase transition. Therefore, data sets were measured between room temperature and 90 K upon cooling and reheating, which shows that a discontinuity in the unit-cell volume occurs between 170 and 180 K. This process is accompanied by the formation of an additional crystal domain. Both domains can be indexed separately, leading to a unit cell similar to that observed at room temperature, but with both α and β angles revealing significant deviations from 90°. Twin refinements clearly show that the structure becomes triclinic (space group *P*1) upon cooling, resulting in two crystallographically independent mol­ecules in the unit cell, for which some structural changes especially in the conformation and arrangement of the mol­ecules are observed. In the low-temperature structure, disordering of the methyl H atoms is still observed. If the crystal is reheated, the reflections of the second domain disappear and the structure can be successfully refined again in the monoclinic space group *P*2_1_/*c*, indicating reversibility of the transition.

## Abstract 2

The title compound undergoes a reversible phase transition upon cooling, during which the space group changes from *P*2_1_/*c* to *P*1, which is accompanied by a discontinuous change in the unit-cell volume. In the low-temperature phase, twinning is observed, which vanishes upon reheating to room temperature.

## 1. Chemical context

Azo-based photoswitchable compounds have attracted significant attention because of their high potential for applications in photopharmacology and smart materials (Burk *et al.*, 2021 ▸; Corrado *et al.*, 2023 ▸; Lancia *et al.*, 2019 ▸; Martino *et al.*, 2020 ▸; Mukherjee *et al.*, 2023 ▸; Li *et al.*, 2023 ▸). The rational design and synthetic accessibility of new compounds with tailored structural and photophysical properties remain key research areas of broad inter­est. While azo­benzenes are well-studied, their ethyl­ene-bridged analogues, known as diazo­cines, exhibit significantly different characteristics. Diazo­cines consist of an azo-containing eight-membered heterocyclic ring (Duval, 1910 ▸; Paudler & Zeiler, 1969 ▸; Perlllmutter, 1990 ▸) and undergo reversible, light-driven isomerization between the thermodynamically stable *Z* form and the metastable *E* form (Moormann *et al.*, 2020 ▸). Compared to azo­benzene, diazo­cines show a bathochromic shift of the irradiation wavelengths required for switching, along with significantly higher *Z* → *E* conversion rates (Siewertsen *et al.*, 2009 ▸, 2011 ▸). Especially in photopharmacology, diazo­cines show huge potential as the steric unfavourable *Z* isomer shows no biological activity and can be reversibly switched to the steric less hindered and biologically active *E* form (Cabré *et al.*, 2019 ▸; Ewert *et al.*, 2022 ▸, López-Cano *et al.*, 2024 ▸). Despite their advantageous properties, the limited availability of efficient synthetic methods for diazo­cine derivatives restricts their broader application. Therefore, the development of new synthetic strategies for the straightforward preparation of suitable diazo­cine-based compounds is essential.

In this context, we have reported on a new modular strategy for the synthesis of such diazo­cine derivatives, based on the late-stage functionalization of a bis-anhydride and a bis-imide of diazo­cine using primary amines or alkyl halides (Businski *et al.*, 2025 ▸). Of twelve newly synthesized bis-*N*-substituted imide diazo­cines, six derivatives were additionally investigated by single-crystal structure determination at low temperatures. However, upon cooling, crystals of the title compound, C_20_H_14_N_4_O_4_ (**1**) show additional reflections that cannot be indexed. Moreover, the diffraction pattern indicates that the crystals develop numerous cracks, presumably due to cooling. To avoid further complications, structure determination was carried out at 220 K, as the diffraction pattern at this temperature corresponds to that of a single crystal. According to this structure determination, compound **1** crystallizes in the monoclinic crystal system, in the centrosymmetric space group *P*2_1_/*c* with *Z* = 4 and one crystallographically independent mol­ecule in a general position.
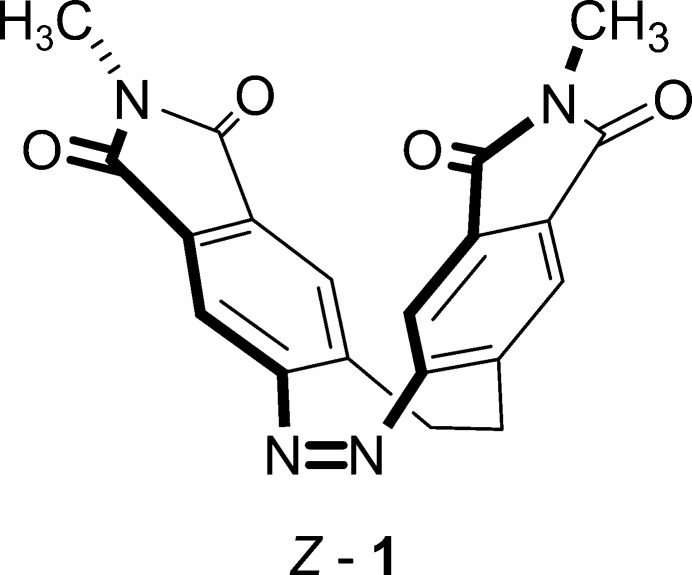


Starting from these results, the crystal structure was investigated in more detail in subsequent work. Therefore, a crystal was slowly cooled, leading to a diffraction pattern that could be successfully indexed by assuming the presence of two twin components. Detailed analysis of these data revealed that the crystal system changes to triclinic (space group *P*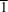), indicating that a phase transition has occurred. Therefore, a series of data sets was collected at various temperatures during both cooling and heating to determine whether the transition is reversible and to observe any associated structural changes.

## 2. Structural commentary

**Figure 1:**
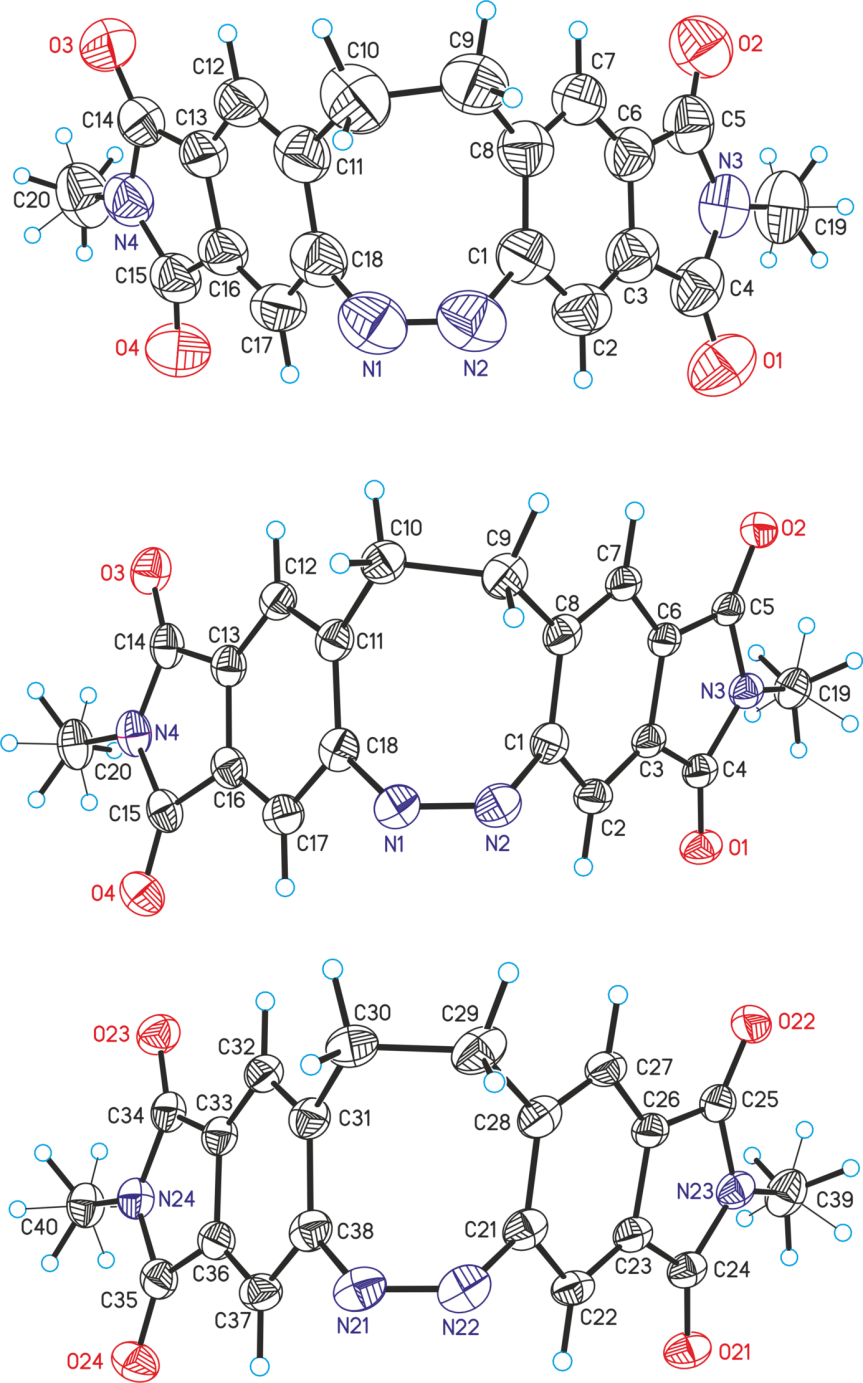
View of the asymmetric unit of **1** at 300 K (top) and at 90 K (middle and bottom) with labeling and displacement ellipsoids drawn at the 50% probability level. Note that in the low-temperature structure, two crystallographically independent mol­ecules are present.

**Figure 2:**
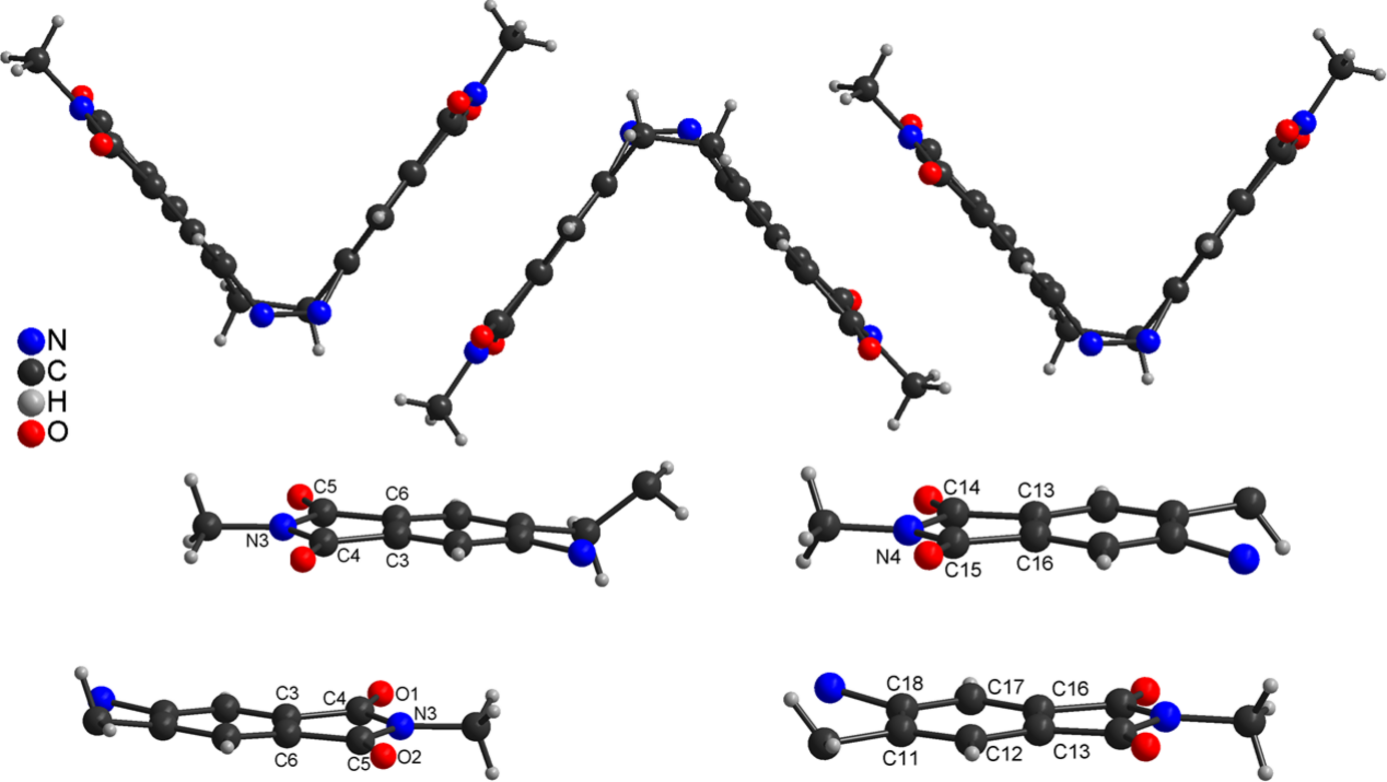
View of the arrangement of neighbouring mol­ecules into chains (top) and relative orientation of the five- and six-membered rings within these chains (bottom) with labeling of selected atoms.

**Figure 3:**
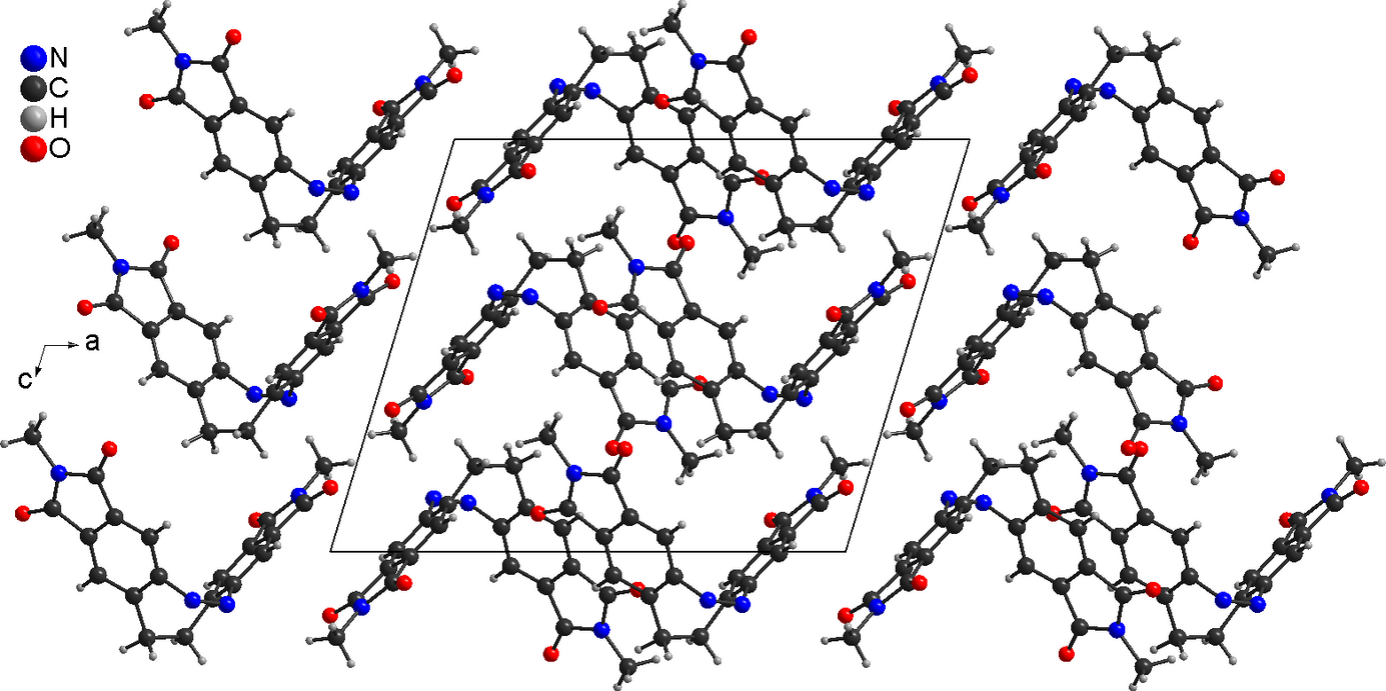
Crystal structure of **1** with view along the crystallographic *b*-axis direction. The disordering of the methyl H atoms is not shown.

As mentioned above, the crystal structure of **1** was already reported at 220 K (Businski *et al.*, 2025 ▸), but for comparison with the low-temperature data, the structure was remeasured at room temperature and is now described in more detail. At this temperature, compound **1** crystallizes in the monoclinic crystal system in the space group *P*2_1_/*c* and *Z* = 4 with one mol­ecule in a general position (Table 1 ▸ and Fig. 1 ▸). As a result of lower ring strain and steric hindrance, the mol­ecule crystallizes in the thermodynamically stable *Z* form with a C—N—N—C torsion angle of −1.7 (2)° (Fig. 1 ▸). The dihedral angle between the best planes calculated for each phthalimide subunit amounts to 78.37 (2)° (Fig. 2 ▸: top). In the crystal structure of **1**, the mol­ecules are arranged in chains that propagate along [110] (Fig. 2 ▸: top). Within these chains, the phthalimide ring planes of neighbouring mol­ecules are parallel, indicating π–π stacking inter­actions (Fig. 2 ▸). In one case, the five-membered rings of adjacent mol­ecules are stacked onto each other with a distance of 3.920 (1) Å between the centroids of the ring planes. In the second case, the five- and six-membered rings inter­act with a centroid–centroid distance of 3.449 (1) Å (Fig. 2 ▸: bottom). Finally, the mol­ecules are arranged into stacks that proceed along the crystallographic *b*-axis direction (Fig. 3 ▸).

## 3. Temperature-dependent measurements and low-temperature structure of **1**

Based on previous results, which indicate that crystals of **1** undergo a phase transition, temperature-dependent measurements were performed between 90 and 300 K upon cooling and reheating.

**Figure 4:**
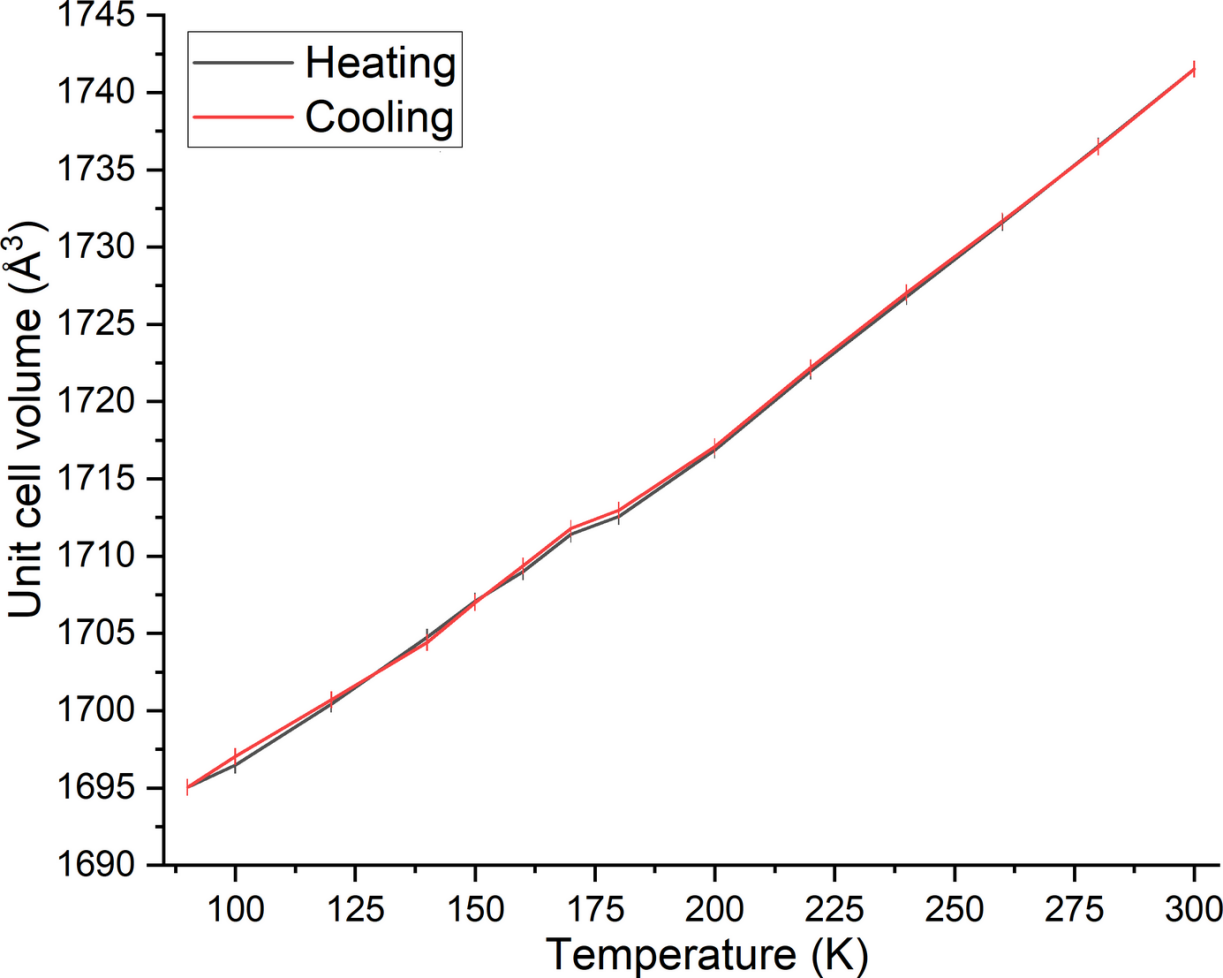
Unit-cell volume of **1** at different temperatures determined in the cooling and heating cycle.

First of all, the unit-cell volume was measured as a function of temperature, initially showing a linear decrease with decreasing temperature down to 180 K. Between 170 and 180 K, an abrupt change of the unit-cell volume takes place and upon further cooling, linear behavior is observed again (Fig. 4 ▸). It is noted that the jump in the unit-cell volume is insignificant within three times of the standard deviation. Surprisingly, the same behavior is observed upon reheating of the crystal, strongly indicating a phase transition. On one hand, a purely first-order phase transition appears unlikely, as the observed behavior seems to be reversible. On the other hand, a second order phase transition can be excluded due to an abrupt change of the unit-cell volume.

**Figure 5:**
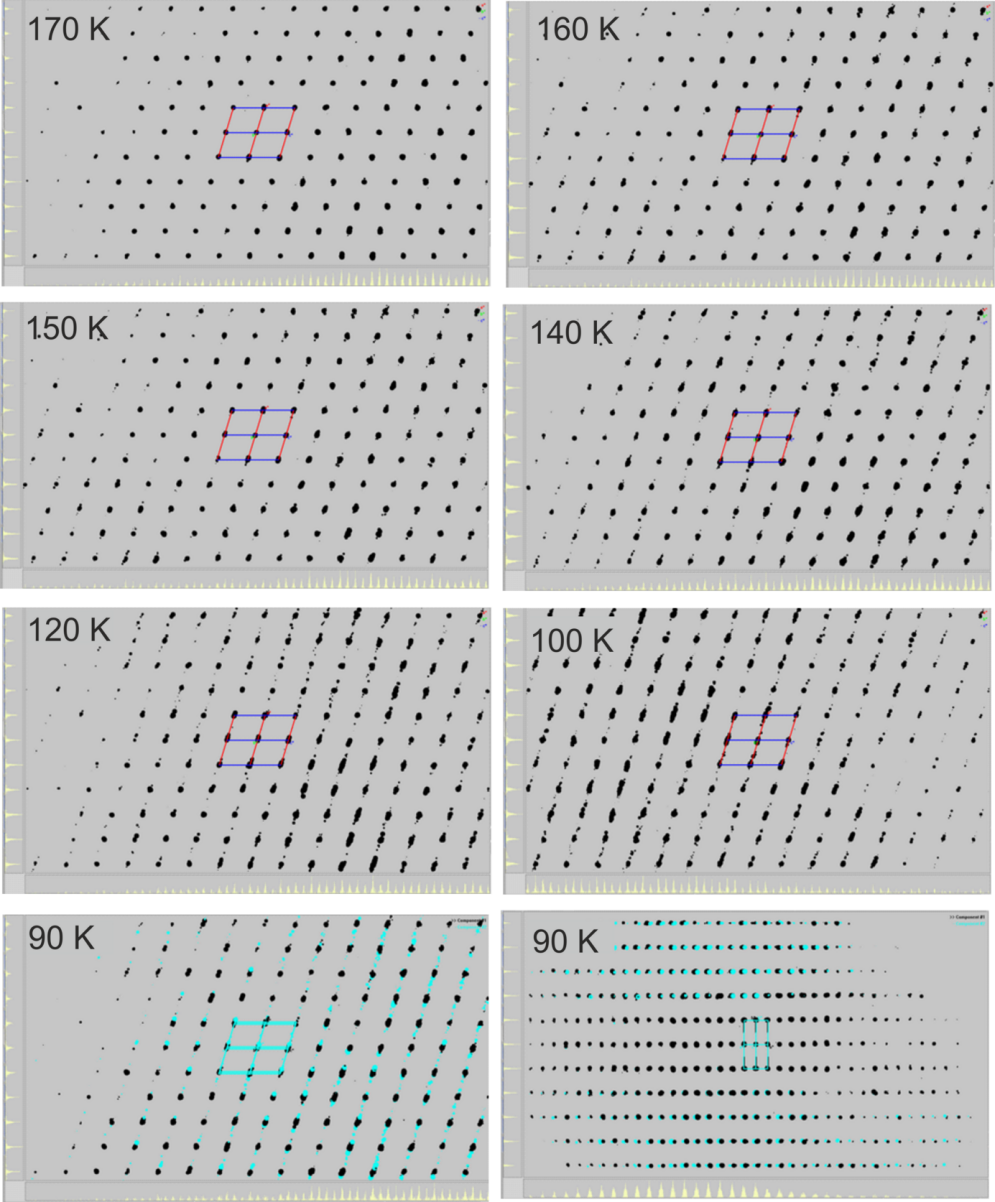
Diffraction pattern of **1** along b* at different temperatures. For the measurement at 90 K, the reflections of both individuals are indicated in black and blue and, additionally, the view along a* is shown (bottom right).

**Figure 6:**
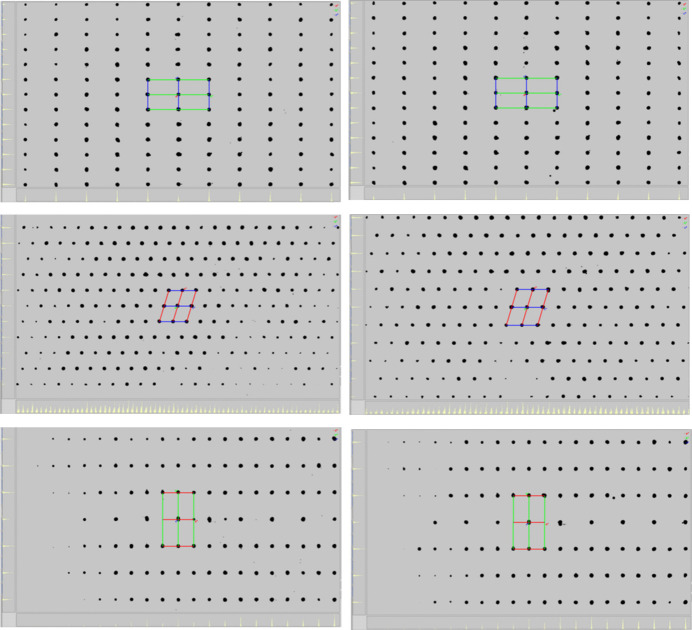
Diffraction pattern of **1** at 260 K upon cooling (left) and reheating (right) with view along a* (top), b* (middle) and c* (bottom).

In the following, the diffraction patterns observed during cooling at different temperatures were analyzed (Fig. 5 ▸). Down to 170 K, the diffraction pattern looks like that of a single crystal. Starting from around 160 K, some reflections show a very small splitting, which is observed in particular at high Bragg angles. This splitting increases with decreasing temperature, and around 140 K it becomes evident that more than one crystal domain is involved. Practically all of these reflections can be indexed, assuming the presence of two twin components, which is shown as an example for the measurement performed at 90 K (Fig. 5 ▸). Indexing leads to a unit cell with all angles different from 90°, for which a triclinic crystal system is suggested (see below). Inter­estingly, upon reheating of this crystal, the second domain disappears and, for example at 260 K, the diffraction pattern corresponds to that of a single crystal once again. It is practically identical to the pattern of the crystal observed at 260 K during the cooling cycle (Fig. 6 ▸). Therefore, this behavior seems to be fully reversible. In this context, it is noted that the number of domains seems to be dependent on the cooling rate and the crystal quality. If a crystal is directly placed into the cooling stream at 100 K, many domains appear and indexing fails.

In subsequent work, numerous refinements were carried out in the monoclinic (space group *P*2_1_/*c*) and triclinic (space group *P*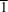) crystal system, either neglecting the twinning or using twin refinements also with data in HKLF-5 format in *SHELXL*. In the beginning, the structure was refined in both space groups using the data obtained at 300 K. In the space group *P*2_1_/*c*, the refinement leads to reasonable reliability factors with no hints for a reduction of the symmetry, which is also obvious from the low inter­nal *R*-value of 1.7%. Nevertheless, these data were also modeled in space group *P*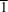, including a twin refinement based on the assumption that the correct symmetry is 2/*m*. This leads to slightly improved *R*-values; however, the BASF parameter refines close to 0.5 and large correlations between the parameters are obtained. Several cycles were needed to reach convergence, clearly proving that the monoclinic symmetry is correct. Moreover, for the structure model in space group *P*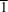, the ADSYMM option in *PLATON* suggests the higher space-group symmetry.

**Figure 7:**
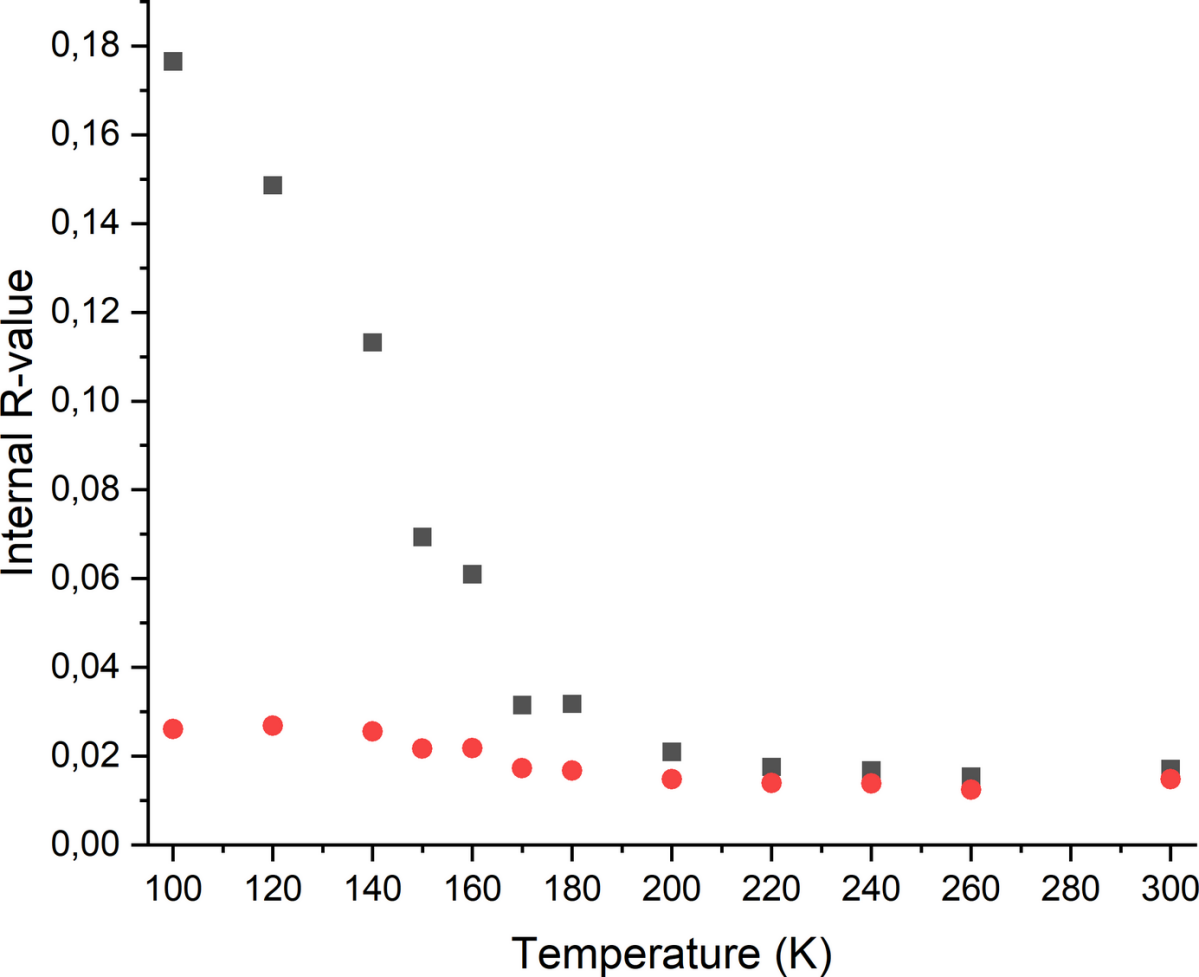
Inter­nal *R*-values obtained for refinements in the monoclinic (black) and triclinic crystal system (red) using data sets measured upon cooling.

If the crystal is cooled down, no changes are observed until 200 K. At 180 K, a slight increase of the inter­nal *R*-value is noticed, which clearly increases upon further cooling. This might also be traced back to the continuous splitting of the reflections, leading to an imprecise measurement of the intensities. However, in the triclinic crystal system, the inter­nal *R*-value remains more or less constant (Fig. 7 ▸). At 160 K, the *R*-value increases dramatically, independent of whether the structure is refined in *P*2_1_/*c* or *P*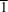, which clearly shows that the splitting of the reflections cannot be neglected any further. Therefore, both individuals were indexed separately and twin refinements using data in HKLF-5 format were performed. In this case, the structure refines much better in the triclinic space group *P*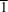 (*R*1 = 0.044 and *wR*2 = 0.127) than in the monoclinic space group *P*2_1_/*c* (*R*1 = 0.085 and *wR*2 = 0.201) indicating that the phase transition is finished. Here, *PLATON* only detects additional pseudo symmetry. Upon further cooling, splitting of the reflections increases and the best resolution is achieved at 90 K. This data set was used for comparison with the high-temperature monoclinic structure.

The low-temperature form of **1** crystallizes in the triclinic space group *P*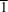 with *Z* = 4 with two crystallographically independent mol­ecules in a general position (Table 1 ▸ and Fig. 1 ▸). The C—N—N—C torsion angles of 3.1 (3)° and −0.4 (3)° in both mol­ecules are only slightly different. Larger changes are observed in the dihedral angles between the phthalimide subunits, which amount to 79.30 (2) and 74.64 (3)° in the low-temperature form, with the latter significantly different from the high-temperature structure. As expected, the overall arrangement of the mol­ecules is similar to that in the high-temperature form. The distances of 3.404 (1) and 3.411 (1) Å between the centroids of the five- and six-membered rings are similar to that in the high-temperature structure [distance = 3.449 (1) Å]. However, larger changes are observed for the distance between the centroids of the five-membered rings, which amount to 3.771 (1) and 3.978 (1) Å, whereas 3.920 (1) Å is found in the high-temperature form (see above). This indicates that the phase transition is accompanied with some mol­ecular movement of the building units.

## 4. Database survey

A search of the CSD (version 5.43, last update December 2024, Groom *et al.*, 2016 ▸) using CONQUEST (Bruno *et al.*, 2002 ▸) reveals that some crystal structures of related compounds with an azo group as part of the central eight-membered ring are reported. These include, *e.g.* (*Z*)-11,12-di­hydro­dibenzo[*c,g*][1,2]diazo­cine (CSD refcode BUYFIL, Siewertsen *et al.*, 2009 ▸; BUYFIL02, Joshi *et al.*, 2012 ▸; BUYFIL03, Kramer *et al.*, 2018 ▸; BUYFIL014, Liu *et al.*, 2023 ▸). Also included are, *e.g.*, *N*,*N*′-(11,12-di­hydro­dibenzo[*c,g*][1,2]diazo­cine-3,8-diylbis(4,1-phenyl­ene)-bis­(*N*-phenyl­aniline) 1,2-dichoro­ethane solvate (EGAPAG, Zhu *et al.*, 2019 ▸) and 3,8-di­bromo­dibenzo[*c,g*][1,2]diazo­cine (GAJMUD, Zhu, 2020 ▸). All of these mol­ecules are in the *Z* form, but there is also an example, where both the *Z* and *E* forms are reported (PEYLEN, Jun *et al.*, 2018 ▸; PEYLEN01, Kramer *et al.*, 2018 ▸; PEYLEN02, Deng *et al.*, 2020 ▸).

## 5. Synthesis and crystallization

**Synthesis**

The synthesis of the title compound was performed according to a procedure reported in the literature (Businski *et al.*, 2025 ▸).

**Crystallization**

The crystals were grown by vapor diffusion experiments using a solvent/anti-solvent mixture of chloro­form and methanol, as also described in the literature (Businski *et al.*, 2025 ▸).

## 6. Refinement

Crystal data, data collection and structure refinement details are summarized in Table 1 ▸. The aromatic H atoms were positioned with idealized geometry and were refined with *U*_iso_(H) = 1.2*U*_eq_(C) using a riding model. The methyl H atoms are disordered and were refined with *U*_iso_(H) = 1.2*U*_eq_(C) in two orientations rotated by each 60° (AFIX 127 card in *SHELXL*) using a riding model. The ratio between the two orientations was also refined. This disorder is also observed in the low-temperature phase and therefore, the same refinement procedure was used. This leads to some differences of the H-atom disorder between the high- and low- temperature phases, but it should be noted that the values for the site occupation factor will not be very reliable, especially at 300 K.

## Supplementary Material

Crystal structure: contains datablock(s) 300K, 90K. DOI: 10.1107/S2056989025003998/hb8139sup1.cif

Structure factors: contains datablock(s) 300K. DOI: 10.1107/S2056989025003998/hb8139300Ksup2.hkl

Structure factors: contains datablock(s) 90K. DOI: 10.1107/S2056989025003998/hb813990Ksup3.hkl

Supporting information file. DOI: 10.1107/S2056989025003998/hb8139300Ksup4.cml

CCDC references: 2448751, 2448750

Additional supporting information:  crystallographic information; 3D view; checkCIF report

## Figures and Tables

**Table 1:** Experimental details

	300 K	90 K
Crystal data		
Chemical formula	C_20_H_14_N_4_O_4_	C_20_H_14_N_4_O_4_
*M*_r_	374.35	374.35
Crystal system, space group	Monoclinic, *P*2_1_/*c*	Triclinic, *P* 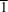
Temperature (K)	300	90
*a*, *b*, *c* (Å)	16.4290 (2), 8.05961 (10), 13.72324 (16)	7.9465 (2), 13.7201 (3), 16.2592 (6)
α, β, γ (°)	90, 106.7581 (14), 90	106.993 (2), 90.914 (3), 90.1121 (19)
*V* (Å^3^)	1739.94 (4)	1695.04 (9)
*Z*	4	4
Radiation type	Cu *K*α	Cu *K*α
μ (mm^−1^)	0.85	0.88
Crystal size (mm)	0.20 × 0.15 × 0.10	0.20 × 0.15 × 0.10

Data collection		
Diffractometer	XtaLAB Synergy, Dualflex, HyPix	XtaLAB Synergy, Dualflex, HyPix
Absorption correction	Multi-scan (*CrysAlis PRO*; Rigaku OD, 2023 ▸)	Multi-scan (*CrysAlis PRO*; Rigaku OD, 2023 ▸)
*T*_min_, *T*_max_	0.875, 1.000	0.915, 1.000
No. of measured, independent and observed [*I* > 2σ(*I*)] reflections	20169, 3738, 3529	7780, 7780, 7595
*R*_int_	0.017	–
(sin θ/λ)_max_ (Å^−1^)	0.639	0.641

Refinement		
*R*[*F*^2^ > 2σ(*F*^2^)], *wR*(*F*^2^), *S*	0.039, 0.109, 1.06	0.041, 0.117, 1.07
No. of reflections	3738	7780
No. of parameters	258	514
H-atom treatment	H-atom parameters constrained	H-atom parameters constrained
Δρ_max_, Δρ_min_ (e Å^−3^)	0.15, −0.16	0.27, −0.20

Computer programs: *CrysAlis PRO* (Rigaku OD, 2023 ▸), *SHELXT2014/5* (Sheldrick, 2015*a* ▸), *SHELXL2016/6* (Sheldrick, 2015*b* ▸), *DIAMOND* (Brandenburg, 1999 ▸) and *XP* in *SHELXTL-PC* (Sheldrick, 2008 ▸), *publCIF* (Westrip, 2010 ▸).

## supplementary crystallographic information

### 8,19-Dimethyl-2,3,8,19-tetraazapentacyclo[13.7.0.04,12.06,10.017,21]docosa-1(15),2,4(12),5,10,16,21-heptaene-7,9,18,20-tetrone (300K) Crystal data

**Table:**

C_20_H_14_N_4_O_4_	*F*(000) = 776
*M**_r_* = 374.35	*D*_x_ = 1.429 Mg m^−^^3^
Monoclinic, *P*2_1_/*c*	Cu *K*α radiation, λ = 1.54184 Å
*a* = 16.4290 (2) Å	Cell parameters from 12821 reflections
*b* = 8.05961 (10) Å	θ = 2.8–78.4°
*c* = 13.72324 (16) Å	µ = 0.85 mm^−^^1^
β = 106.7581 (14)°	*T* = 300 K
*V* = 1739.94 (4) Å^3^	Block, colourless
*Z* = 4	0.20 × 0.15 × 0.10 mm

### 8,19-Dimethyl-2,3,8,19-tetraazapentacyclo[13.7.0.04,12.06,10.017,21]docosa-1(15),2,4(12),5,10,16,21-heptaene-7,9,18,20-tetrone (300K) Data collection

**Table:**

XtaLAB Synergy, Dualflex, HyPix diffractometer	3738 independent reflections
Radiation source: micro-focus sealed X-ray tube, PhotonJet (Cu) X-ray Source	3529 reflections with *I* > 2σ(*I*)
Mirror monochromator	*R*_int_ = 0.017
Detector resolution: 10.0000 pixels mm^-1^	θ_max_ = 80.3°, θ_min_ = 2.8°
ω scans	*h* = −20→20
Absorption correction: multi-scan (CrysAlisPro; Rigaku OD, 2023)	*k* = −10→10
*T*_min_ = 0.875, *T*_max_ = 1.000	*l* = −16→9
20169 measured reflections	

### 8,19-Dimethyl-2,3,8,19-tetraazapentacyclo[13.7.0.04,12.06,10.017,21]docosa-1(15),2,4(12),5,10,16,21-heptaene-7,9,18,20-tetrone (300K) Refinement

**Table:**

Refinement on *F*^2^	Hydrogen site location: inferred from neighbouring sites
Least-squares matrix: full	H-atom parameters constrained
*R*[*F*^2^ > 2σ(*F*^2^)] = 0.039	*w* = 1/[σ^2^(*F*_o_^2^) + (0.0523*P*)^2^ + 0.2804*P*] where *P* = (*F*_o_^2^ + 2*F*_c_^2^)/3
*wR*(*F*^2^) = 0.109	(Δ/σ)_max_ < 0.001
*S* = 1.06	Δρ_max_ = 0.15 e Å^−^^3^
3738 reflections	Δρ_min_ = −0.16 e Å^−^^3^
258 parameters	Extinction correction: *SHELXL2016/6* (Sheldrick, 2015b)), Fc^*^=kFc[1+0.001xFc^2^λ^3^/sin(2θ)]^-1/4^
0 restraints	Extinction coefficient: 0.0017 (2)
Primary atom site location: dual	

### 8,19-Dimethyl-2,3,8,19-tetraazapentacyclo[13.7.0.04,12.06,10.017,21]docosa-1(15),2,4(12),5,10,16,21-heptaene-7,9,18,20-tetrone (300K) Special details

**Table:**

Geometry. All esds (except the esd in the dihedral angle between two l.s. planes) are estimated using the full covariance matrix. The cell esds are taken into account individually in the estimation of esds in distances, angles and torsion angles; correlations between esds in cell parameters are only used when they are defined by crystal symmetry. An approximate (isotropic) treatment of cell esds is used for estimating esds involving l.s. planes.

### 8,19-Dimethyl-2,3,8,19-tetraazapentacyclo[13.7.0.04,12.06,10.017,21]docosa-1(15),2,4(12),5,10,16,21-heptaene-7,9,18,20-tetrone (300K) Fractional atomic coordinates and isotropic or equivalent isotropic displacement parameters (Å^2^)

**Table:**

	*x*	*y*	*z*	*U*_iso_*/*U*_eq_	Occ. (<1)
N1	0.76123 (9)	0.02530 (15)	0.61812 (9)	0.0675 (3)	
N2	0.83005 (8)	0.09831 (15)	0.62847 (9)	0.0662 (3)	
C1	0.83034 (8)	0.25742 (16)	0.57982 (10)	0.0556 (3)	
C2	0.87140 (8)	0.25936 (17)	0.50414 (10)	0.0593 (3)	
H2	0.893926	0.163217	0.484767	0.071*	
C3	0.87714 (8)	0.40923 (17)	0.45949 (10)	0.0568 (3)	
C4	0.92117 (8)	0.4547 (2)	0.38251 (10)	0.0643 (3)	
O1	0.96289 (8)	0.36729 (17)	0.34456 (9)	0.0866 (4)	
N3	0.90683 (7)	0.62318 (17)	0.36506 (9)	0.0677 (3)	
C5	0.86000 (9)	0.69145 (19)	0.42526 (11)	0.0629 (3)	
O2	0.83808 (8)	0.83548 (14)	0.42375 (9)	0.0825 (3)	
C6	0.84279 (8)	0.55260 (16)	0.48743 (10)	0.0552 (3)	
C7	0.80432 (8)	0.55111 (16)	0.56413 (10)	0.0554 (3)	
H7	0.781944	0.648116	0.582664	0.067*	
C8	0.79932 (8)	0.40161 (16)	0.61389 (10)	0.0549 (3)	
C9	0.76753 (10)	0.40375 (19)	0.70657 (11)	0.0660 (4)	
H9A	0.750474	0.516544	0.715700	0.079*	
H9B	0.815176	0.377276	0.765100	0.079*	
C10	0.69453 (12)	0.2901 (2)	0.70905 (11)	0.0746 (4)	
H10A	0.717056	0.197918	0.754194	0.090*	
H10B	0.655912	0.350875	0.737728	0.090*	
C11	0.64478 (9)	0.22184 (16)	0.60706 (10)	0.0579 (3)	
C12	0.56314 (9)	0.27668 (15)	0.55744 (11)	0.0582 (3)	
H12	0.536512	0.355374	0.587290	0.070*	
C13	0.52247 (8)	0.21175 (14)	0.46287 (10)	0.0525 (3)	
C14	0.43587 (9)	0.24216 (15)	0.39487 (12)	0.0596 (3)	
O3	0.38113 (7)	0.33220 (13)	0.40784 (10)	0.0801 (3)	
N4	0.42771 (7)	0.14206 (13)	0.31000 (9)	0.0608 (3)	
C15	0.50120 (9)	0.05343 (15)	0.31613 (10)	0.0557 (3)	
O4	0.51166 (8)	−0.03756 (14)	0.25120 (8)	0.0753 (3)	
C16	0.56139 (8)	0.09568 (14)	0.41654 (9)	0.0502 (3)	
C17	0.64128 (8)	0.03570 (15)	0.46471 (10)	0.0540 (3)	
H17	0.666937	−0.044650	0.434837	0.065*	
C18	0.68183 (9)	0.10091 (15)	0.56018 (10)	0.0551 (3)	
C19	0.93771 (11)	0.7171 (3)	0.29226 (13)	0.0833 (5)	
H19A	0.951253	0.828131	0.316967	0.125*	0.74 (3)
H19B	0.894512	0.720415	0.227977	0.125*	0.74 (3)
H19C	0.987726	0.664703	0.283848	0.125*	0.74 (3)
H19D	0.937741	0.647368	0.235561	0.125*	0.26 (3)
H19E	0.994482	0.755084	0.324552	0.125*	0.26 (3)
H19F	0.901268	0.810796	0.268680	0.125*	0.26 (3)
C20	0.35482 (11)	0.1457 (2)	0.22019 (14)	0.0822 (5)	
H20A	0.363199	0.067773	0.171044	0.123*	0.60 (2)
H20B	0.348438	0.255177	0.191437	0.123*	0.60 (2)
H20C	0.304558	0.116489	0.238622	0.123*	0.60 (2)
H20D	0.314264	0.225187	0.229692	0.123*	0.40 (2)
H20E	0.329025	0.037782	0.209298	0.123*	0.40 (2)
H20F	0.372905	0.176470	0.162113	0.123*	0.40 (2)

### 8,19-Dimethyl-2,3,8,19-tetraazapentacyclo[13.7.0.04,12.06,10.017,21]docosa-1(15),2,4(12),5,10,16,21-heptaene-7,9,18,20-tetrone (300K) Atomic displacement parameters (Å^2^)

**Table:**

	*U*^11^	*U*^22^	*U*^33^	*U*^12^	*U*^13^	*U*^23^
N1	0.0796 (8)	0.0536 (6)	0.0645 (7)	0.0053 (6)	0.0130 (6)	0.0104 (5)
N2	0.0737 (7)	0.0571 (6)	0.0604 (6)	0.0085 (6)	0.0077 (5)	0.0060 (5)
C1	0.0542 (6)	0.0539 (7)	0.0526 (6)	0.0041 (5)	0.0054 (5)	0.0010 (5)
C2	0.0562 (7)	0.0596 (7)	0.0575 (7)	0.0090 (6)	0.0092 (5)	−0.0054 (6)
C3	0.0486 (6)	0.0665 (8)	0.0519 (6)	0.0022 (5)	0.0089 (5)	−0.0039 (6)
C4	0.0516 (7)	0.0834 (10)	0.0546 (7)	0.0025 (6)	0.0100 (5)	−0.0017 (6)
O1	0.0828 (7)	0.1095 (9)	0.0754 (7)	0.0168 (7)	0.0351 (6)	−0.0025 (6)
N3	0.0574 (6)	0.0818 (8)	0.0623 (7)	−0.0063 (6)	0.0150 (5)	0.0083 (6)
C5	0.0549 (7)	0.0668 (8)	0.0626 (8)	−0.0044 (6)	0.0097 (6)	0.0042 (6)
O2	0.0947 (8)	0.0632 (7)	0.0909 (8)	−0.0015 (6)	0.0286 (6)	0.0118 (5)
C6	0.0481 (6)	0.0578 (7)	0.0557 (7)	−0.0009 (5)	0.0087 (5)	−0.0002 (5)
C7	0.0535 (6)	0.0522 (7)	0.0587 (7)	0.0013 (5)	0.0130 (5)	−0.0044 (5)
C8	0.0530 (6)	0.0566 (7)	0.0517 (6)	0.0003 (5)	0.0094 (5)	−0.0029 (5)
C9	0.0779 (9)	0.0662 (8)	0.0536 (7)	−0.0005 (7)	0.0184 (6)	−0.0073 (6)
C10	0.1017 (12)	0.0747 (9)	0.0539 (7)	−0.0155 (8)	0.0325 (8)	−0.0054 (7)
C11	0.0746 (8)	0.0510 (7)	0.0556 (7)	−0.0089 (6)	0.0308 (6)	−0.0002 (5)
C12	0.0724 (8)	0.0461 (6)	0.0684 (8)	−0.0062 (5)	0.0400 (7)	−0.0063 (5)
C13	0.0609 (7)	0.0393 (5)	0.0661 (7)	−0.0025 (5)	0.0323 (6)	0.0019 (5)
C14	0.0608 (7)	0.0418 (6)	0.0836 (9)	−0.0028 (5)	0.0325 (7)	0.0048 (6)
O3	0.0656 (6)	0.0598 (6)	0.1233 (10)	0.0078 (5)	0.0404 (6)	−0.0016 (6)
N4	0.0606 (6)	0.0488 (5)	0.0719 (7)	−0.0019 (5)	0.0175 (5)	0.0075 (5)
C15	0.0670 (7)	0.0454 (6)	0.0584 (7)	−0.0021 (5)	0.0240 (6)	0.0042 (5)
O4	0.0898 (7)	0.0739 (7)	0.0622 (6)	0.0046 (5)	0.0221 (5)	−0.0124 (5)
C16	0.0616 (7)	0.0402 (5)	0.0550 (6)	−0.0014 (5)	0.0265 (5)	0.0022 (5)
C17	0.0659 (7)	0.0433 (6)	0.0582 (7)	0.0037 (5)	0.0263 (6)	0.0006 (5)
C18	0.0669 (7)	0.0456 (6)	0.0548 (6)	−0.0015 (5)	0.0209 (5)	0.0066 (5)
C19	0.0714 (9)	0.1092 (14)	0.0681 (9)	−0.0140 (9)	0.0182 (7)	0.0176 (9)
C20	0.0725 (9)	0.0713 (9)	0.0904 (11)	−0.0076 (8)	0.0038 (8)	0.0153 (8)

### 8,19-Dimethyl-2,3,8,19-tetraazapentacyclo[13.7.0.04,12.06,10.017,21]docosa-1(15),2,4(12),5,10,16,21-heptaene-7,9,18,20-tetrone (300K) Geometric parameters (Å, º)

**Table:**

N1—N2	1.2458 (17)	C11—C18	1.4000 (18)
N1—C18	1.4516 (18)	C12—H12	0.9300
N2—C1	1.4464 (17)	C12—C13	1.3800 (19)
C1—C2	1.3920 (19)	C13—C14	1.4797 (19)
C1—C8	1.4023 (18)	C13—C16	1.3866 (16)
C2—H2	0.9300	C14—O3	1.2080 (16)
C2—C3	1.370 (2)	C14—N4	1.3911 (19)
C3—C4	1.4881 (19)	N4—C15	1.3844 (17)
C3—C6	1.3876 (18)	N4—C20	1.450 (2)
C4—O1	1.2012 (18)	C15—O4	1.2039 (16)
C4—N3	1.387 (2)	C15—C16	1.4855 (18)
N3—C5	1.3942 (19)	C16—C17	1.3759 (18)
N3—C19	1.4564 (19)	C17—H17	0.9300
C5—O2	1.2138 (18)	C17—C18	1.3904 (18)
C5—C6	1.4830 (19)	C19—H19A	0.9600
C6—C7	1.3752 (19)	C19—H19B	0.9600
C7—H7	0.9300	C19—H19C	0.9600
C7—C8	1.3990 (18)	C19—H19D	0.9600
C8—C9	1.5077 (19)	C19—H19E	0.9600
C9—H9A	0.9700	C19—H19F	0.9600
C9—H9B	0.9700	C20—H20A	0.9600
C9—C10	1.517 (2)	C20—H20B	0.9600
C10—H10A	0.9700	C20—H20C	0.9600
C10—H10B	0.9700	C20—H20D	0.9600
C10—C11	1.507 (2)	C20—H20E	0.9600
C11—C12	1.390 (2)	C20—H20F	0.9600
			
N2—N1—C18	120.45 (11)	C15—N4—C20	123.64 (13)
N1—N2—C1	119.33 (11)	N4—C15—C16	105.88 (11)
C2—C1—N2	114.91 (12)	O4—C15—N4	125.27 (13)
C2—C1—C8	122.71 (12)	O4—C15—C16	128.85 (13)
C8—C1—N2	122.07 (12)	C13—C16—C15	107.96 (11)
C1—C2—H2	121.5	C17—C16—C13	121.25 (12)
C3—C2—C1	117.02 (12)	C17—C16—C15	130.75 (11)
C3—C2—H2	121.5	C16—C17—H17	121.6
C2—C3—C4	130.16 (13)	C16—C17—C18	116.84 (11)
C2—C3—C6	121.65 (12)	C18—C17—H17	121.6
C6—C3—C4	108.11 (12)	C11—C18—N1	118.79 (12)
O1—C4—C3	128.19 (15)	C17—C18—N1	117.92 (12)
O1—C4—N3	126.08 (15)	C17—C18—C11	122.85 (13)
N3—C4—C3	105.69 (12)	N3—C19—H19A	109.5
C4—N3—C5	112.29 (12)	N3—C19—H19B	109.5
C4—N3—C19	123.49 (14)	N3—C19—H19C	109.5
C5—N3—C19	124.21 (15)	N3—C19—H19D	109.5
N3—C5—C6	105.81 (12)	N3—C19—H19E	109.5
O2—C5—N3	125.38 (14)	N3—C19—H19F	109.5
O2—C5—C6	128.80 (14)	H19A—C19—H19B	109.5
C3—C6—C5	108.01 (12)	H19A—C19—H19C	109.5
C7—C6—C3	121.17 (12)	H19A—C19—H19D	141.1
C7—C6—C5	130.76 (12)	H19A—C19—H19E	56.3
C6—C7—H7	120.5	H19A—C19—H19F	56.3
C6—C7—C8	119.06 (12)	H19B—C19—H19C	109.5
C8—C7—H7	120.5	H19B—C19—H19D	56.3
C1—C8—C9	122.57 (12)	H19B—C19—H19E	141.1
C7—C8—C1	118.22 (12)	H19B—C19—H19F	56.3
C7—C8—C9	119.03 (12)	H19C—C19—H19D	56.3
C8—C9—H9A	107.6	H19C—C19—H19E	56.3
C8—C9—H9B	107.6	H19C—C19—H19F	141.1
C8—C9—C10	118.71 (12)	H19D—C19—H19E	109.5
H9A—C9—H9B	107.1	H19D—C19—H19F	109.5
C10—C9—H9A	107.6	H19E—C19—H19F	109.5
C10—C9—H9B	107.6	N4—C20—H20A	109.5
C9—C10—H10A	108.6	N4—C20—H20B	109.5
C9—C10—H10B	108.6	N4—C20—H20C	109.5
H10A—C10—H10B	107.5	N4—C20—H20D	109.5
C11—C10—C9	114.84 (11)	N4—C20—H20E	109.5
C11—C10—H10A	108.6	N4—C20—H20F	109.5
C11—C10—H10B	108.6	H20A—C20—H20B	109.5
C12—C11—C10	121.83 (13)	H20A—C20—H20C	109.5
C12—C11—C18	118.83 (12)	H20A—C20—H20D	141.1
C18—C11—C10	119.34 (14)	H20A—C20—H20E	56.3
C11—C12—H12	120.7	H20A—C20—H20F	56.3
C13—C12—C11	118.52 (12)	H20B—C20—H20C	109.5
C13—C12—H12	120.7	H20B—C20—H20D	56.3
C12—C13—C14	130.32 (12)	H20B—C20—H20E	141.1
C12—C13—C16	121.65 (12)	H20B—C20—H20F	56.3
C16—C13—C14	108.01 (11)	H20C—C20—H20D	56.3
O3—C14—C13	128.89 (14)	H20C—C20—H20E	56.3
O3—C14—N4	125.15 (14)	H20C—C20—H20F	141.1
N4—C14—C13	105.96 (11)	H20D—C20—H20E	109.5
C14—N4—C20	123.81 (13)	H20D—C20—H20F	109.5
C15—N4—C14	112.12 (11)	H20E—C20—H20F	109.5
			
N1—N2—C1—C2	−114.90 (15)	C9—C10—C11—C12	−107.59 (16)
N1—N2—C1—C8	71.39 (17)	C9—C10—C11—C18	71.79 (18)
N2—N1—C18—C11	−80.49 (17)	C10—C11—C12—C13	178.17 (12)
N2—N1—C18—C17	106.88 (15)	C10—C11—C18—N1	9.94 (18)
N2—C1—C2—C3	−176.74 (11)	C10—C11—C18—C17	−177.82 (12)
N2—C1—C8—C7	178.19 (12)	C11—C12—C13—C14	177.76 (11)
N2—C1—C8—C9	3.17 (19)	C11—C12—C13—C16	−0.71 (18)
C1—C2—C3—C4	175.54 (12)	C12—C11—C18—N1	−170.67 (11)
C1—C2—C3—C6	−0.71 (19)	C12—C11—C18—C17	1.58 (18)
C1—C8—C9—C10	−58.56 (19)	C12—C13—C14—O3	0.0 (2)
C2—C1—C8—C7	4.97 (19)	C12—C13—C14—N4	−179.03 (12)
C2—C1—C8—C9	−170.05 (12)	C12—C13—C16—C15	−179.44 (11)
C2—C3—C4—O1	−1.6 (2)	C12—C13—C16—C17	2.41 (18)
C2—C3—C4—N3	−179.52 (13)	C13—C14—N4—C15	−1.29 (13)
C2—C3—C6—C5	179.92 (11)	C13—C14—N4—C20	−174.09 (12)
C2—C3—C6—C7	2.54 (19)	C13—C16—C17—C18	−2.00 (17)
C3—C4—N3—C5	1.69 (15)	C14—C13—C16—C15	1.79 (13)
C3—C4—N3—C19	−178.53 (13)	C14—C13—C16—C17	−176.37 (10)
C3—C6—C7—C8	−0.57 (18)	C14—N4—C15—O4	−177.35 (13)
C4—C3—C6—C5	2.94 (14)	C14—N4—C15—C16	2.35 (13)
C4—C3—C6—C7	−174.45 (11)	O3—C14—N4—C15	179.61 (12)
C4—N3—C5—O2	−179.05 (14)	O3—C14—N4—C20	6.8 (2)
C4—N3—C5—C6	0.05 (15)	N4—C15—C16—C13	−2.53 (13)
O1—C4—N3—C5	−176.34 (14)	N4—C15—C16—C17	175.38 (12)
O1—C4—N3—C19	3.4 (2)	C15—C16—C17—C18	−179.68 (11)
N3—C5—C6—C3	−1.92 (14)	O4—C15—C16—C13	177.15 (13)
N3—C5—C6—C7	175.13 (13)	O4—C15—C16—C17	−4.9 (2)
C5—C6—C7—C8	−177.28 (13)	C16—C13—C14—O3	178.66 (13)
O2—C5—C6—C3	177.14 (15)	C16—C13—C14—N4	−0.40 (13)
O2—C5—C6—C7	−5.8 (2)	C16—C17—C18—N1	172.34 (11)
C6—C3—C4—O1	175.09 (14)	C16—C17—C18—C11	0.03 (18)
C6—C3—C4—N3	−2.88 (14)	C18—N1—N2—C1	−1.7 (2)
C6—C7—C8—C1	−3.02 (18)	C18—C11—C12—C13	−1.21 (17)
C6—C7—C8—C9	172.18 (12)	C19—N3—C5—O2	1.2 (2)
C7—C8—C9—C10	126.47 (15)	C19—N3—C5—C6	−179.73 (13)
C8—C1—C2—C3	−3.08 (19)	C20—N4—C15—O4	−4.5 (2)
C8—C9—C10—C11	−16.6 (2)	C20—N4—C15—C16	175.16 (12)

### (90K) Crystal data

**Table:**

C_20_H_14_N_4_O_4_	*Z* = 4
*M**_r_* = 374.35	*F*(000) = 776
Triclinic, *P*1	*D*_x_ = 1.467 Mg m^−^^3^
*a* = 7.9465 (2) Å	Cu *K*α radiation, λ = 1.54184 Å
*b* = 13.7201 (3) Å	Cell parameters from 13461 reflections
*c* = 16.2592 (6) Å	θ = 2.8–79.6°
α = 106.993 (2)°	µ = 0.88 mm^−^^1^
β = 90.914 (3)°	*T* = 90 K
γ = 90.1121 (19)°	Block, colourless
*V* = 1695.04 (9) Å^3^	0.20 × 0.15 × 0.10 mm

### (90K) Data collection

**Table:**

XtaLAB Synergy, Dualflex, HyPix diffractometer	7780 measured reflections
Radiation source: micro-focus sealed X-ray tube, PhotonJet (Cu) X-ray Source	7780 independent reflections
Mirror monochromator	7595 reflections with *I* > 2σ(*I*)
Detector resolution: 10.0000 pixels mm^-1^	θ_max_ = 81.0°, θ_min_ = 2.8°
ω scans	*h* = −9→9
Absorption correction: multi-scan (CrysAlisPro; Rigaku OD, 2023)	*k* = −16→16
*T*_min_ = 0.915, *T*_max_ = 1.000	*l* = −20→20

### (90K) Refinement

**Table:**

Refinement on *F*^2^	0 restraints
Least-squares matrix: full	Hydrogen site location: inferred from neighbouring sites
*R*[*F*^2^ > 2σ(*F*^2^)] = 0.041	H-atom parameters constrained
*wR*(*F*^2^) = 0.117	*w* = 1/[σ^2^(*F*_o_^2^) + (0.064*P*)^2^ + 0.6702*P*] where *P* = (*F*_o_^2^ + 2*F*_c_^2^)/3
*S* = 1.07	(Δ/σ)_max_ < 0.001
7780 reflections	Δρ_max_ = 0.27 e Å^−^^3^
514 parameters	Δρ_min_ = −0.20 e Å^−^^3^

### (90K) Special details

**Table:**

Geometry. All esds (except the esd in the dihedral angle between two l.s. planes) are estimated using the full covariance matrix. The cell esds are taken into account individually in the estimation of esds in distances, angles and torsion angles; correlations between esds in cell parameters are only used when they are defined by crystal symmetry. An approximate (isotropic) treatment of cell esds is used for estimating esds involving l.s. planes.
Refinement. Refined as a 2-component twin.

### (90K) Fractional atomic coordinates and isotropic or equivalent isotropic displacement parameters (Å^2^)

**Table:**

	*x*	*y*	*z*	*U*_iso_*/*U*_eq_	Occ. (<1)
N1	0.90321 (19)	0.62743 (12)	0.84068 (10)	0.0300 (3)	
N2	0.97717 (19)	0.61742 (13)	0.77174 (11)	0.0309 (3)	
C1	0.8973 (2)	0.56223 (14)	0.69017 (11)	0.0261 (3)	
C2	0.9597 (2)	0.46667 (14)	0.64684 (11)	0.0262 (3)	
H2	1.039969	0.433158	0.672823	0.031*	
C3	0.89879 (19)	0.42282 (13)	0.56379 (11)	0.0237 (3)	
C4	0.9396 (2)	0.32410 (13)	0.49971 (11)	0.0249 (3)	
O1	1.02862 (16)	0.25596 (10)	0.50834 (9)	0.0317 (3)	
N3	0.85302 (18)	0.32384 (12)	0.42458 (10)	0.0260 (3)	
C5	0.75329 (19)	0.40966 (13)	0.43556 (11)	0.0246 (3)	
O2	0.66287 (16)	0.42633 (11)	0.38013 (8)	0.0308 (3)	
C6	0.78443 (19)	0.47356 (13)	0.52580 (11)	0.0236 (3)	
C7	0.7216 (2)	0.56791 (14)	0.56909 (11)	0.0251 (3)	
H7	0.642211	0.601045	0.542300	0.030*	
C8	0.7781 (2)	0.61345 (13)	0.65364 (12)	0.0263 (3)	
C9	0.7141 (2)	0.71498 (14)	0.70747 (13)	0.0312 (4)	
H9A	0.660111	0.750500	0.669137	0.037*	
H9B	0.810885	0.757169	0.736927	0.037*	
C10	0.5865 (2)	0.70611 (15)	0.77551 (12)	0.0305 (4)	
H10A	0.601282	0.766655	0.826239	0.037*	
H10B	0.472099	0.709803	0.751648	0.037*	
C11	0.5930 (2)	0.61246 (14)	0.80677 (11)	0.0267 (4)	
C12	0.4413 (2)	0.56123 (14)	0.81024 (11)	0.0269 (3)	
H12	0.340053	0.579717	0.786947	0.032*	
C13	0.4425 (2)	0.48339 (14)	0.84824 (11)	0.0274 (4)	
C14	0.3018 (2)	0.41984 (15)	0.86444 (11)	0.0304 (4)	
O3	0.15479 (17)	0.41887 (12)	0.84211 (9)	0.0372 (3)	
N4	0.3728 (2)	0.35902 (13)	0.91086 (10)	0.0327 (3)	
C15	0.5451 (3)	0.37703 (15)	0.92671 (11)	0.0307 (4)	
O4	0.63512 (19)	0.33915 (11)	0.96906 (9)	0.0379 (3)	
C16	0.5881 (2)	0.45571 (14)	0.88382 (11)	0.0283 (4)	
C17	0.7409 (2)	0.50202 (14)	0.87961 (11)	0.0289 (4)	
H17	0.841233	0.482642	0.903060	0.035*	
C18	0.7401 (2)	0.57893 (14)	0.83902 (11)	0.0276 (4)	
C19	0.8451 (2)	0.23684 (15)	0.34772 (12)	0.0331 (4)	
H19A	0.928068	0.186076	0.352851	0.050*	0.93 (3)
H19B	0.869872	0.259322	0.297236	0.050*	0.93 (3)
H19C	0.732144	0.206504	0.341281	0.050*	0.93 (3)
H19D	0.758655	0.248525	0.308061	0.050*	0.07 (3)
H19E	0.816850	0.175279	0.363676	0.050*	0.07 (3)
H19F	0.954579	0.228098	0.319631	0.050*	0.07 (3)
C20	0.2788 (3)	0.28521 (16)	0.94091 (13)	0.0383 (5)	
H20A	0.163081	0.308920	0.953165	0.057*	0.84 (3)
H20B	0.332539	0.277794	0.993401	0.057*	0.84 (3)
H20C	0.277532	0.219228	0.896377	0.057*	0.84 (3)
H20D	0.352353	0.228375	0.942130	0.057*	0.16 (3)
H20E	0.182896	0.259500	0.901894	0.057*	0.16 (3)
H20F	0.237903	0.318067	0.998918	0.057*	0.16 (3)
N21	0.5872 (2)	1.13833 (13)	0.82254 (11)	0.0329 (3)	
N22	0.5151 (2)	1.12574 (13)	0.75096 (11)	0.0343 (4)	
C21	0.5926 (2)	1.06246 (14)	0.67341 (12)	0.0278 (4)	
C22	0.5259 (2)	0.96536 (14)	0.63597 (12)	0.0266 (3)	
H22	0.440615	0.937784	0.663066	0.032*	
C23	0.5897 (2)	0.91128 (13)	0.55773 (12)	0.0250 (3)	
C24	0.5478 (2)	0.80709 (14)	0.50087 (12)	0.0269 (4)	
O21	0.45339 (16)	0.74395 (10)	0.51439 (9)	0.0334 (3)	
N23	0.64090 (18)	0.79380 (12)	0.42735 (10)	0.0289 (3)	
C25	0.7447 (2)	0.87790 (15)	0.43250 (12)	0.0290 (4)	
O22	0.83897 (16)	0.88526 (12)	0.37710 (9)	0.0366 (3)	
C26	0.7106 (2)	0.95301 (14)	0.51710 (12)	0.0258 (3)	
C27	0.7773 (2)	1.04919 (15)	0.55469 (12)	0.0286 (4)	
H27	0.861235	1.076412	0.526518	0.034*	
C28	0.7189 (2)	1.10525 (14)	0.63476 (13)	0.0294 (4)	
C29	0.7888 (3)	1.20923 (15)	0.68072 (15)	0.0372 (4)	
H29A	0.694153	1.257677	0.695896	0.045*	
H29B	0.861377	1.231700	0.640681	0.045*	
C30	0.8919 (2)	1.21495 (14)	0.76328 (13)	0.0329 (4)	
H30A	1.009959	1.231213	0.753442	0.040*	
H30B	0.849053	1.272850	0.809963	0.040*	
C31	0.8929 (2)	1.12217 (14)	0.79500 (11)	0.0279 (4)	
C32	1.0444 (2)	1.07131 (14)	0.80001 (11)	0.0277 (4)	
H32	1.144643	1.089582	0.776876	0.033*	
C33	1.0454 (2)	0.99457 (14)	0.83895 (11)	0.0271 (4)	
C34	1.1855 (2)	0.93178 (15)	0.85713 (11)	0.0295 (4)	
O23	1.33179 (16)	0.92995 (12)	0.83575 (9)	0.0360 (3)	
N24	1.1167 (2)	0.87185 (13)	0.90418 (10)	0.0307 (3)	
C35	0.9451 (2)	0.89021 (14)	0.91788 (11)	0.0301 (4)	
O24	0.85636 (19)	0.85188 (12)	0.95972 (9)	0.0388 (3)	
C36	0.8996 (2)	0.96745 (14)	0.87349 (11)	0.0276 (4)	
C37	0.7477 (2)	1.01241 (14)	0.86708 (12)	0.0291 (4)	
H37	0.647957	0.992735	0.889717	0.035*	
C38	0.7466 (2)	1.08832 (14)	0.82577 (12)	0.0280 (4)	
C39	0.6458 (3)	0.69911 (17)	0.35796 (14)	0.0400 (5)	
H39A	0.561599	0.651555	0.367721	0.060*	0.48 (3)
H39B	0.757924	0.669068	0.356180	0.060*	0.48 (3)
H39C	0.621057	0.712784	0.303147	0.060*	0.48 (3)
H39D	0.732122	0.704049	0.316978	0.060*	0.52 (3)
H39E	0.535796	0.686536	0.328519	0.060*	0.52 (3)
H39F	0.672663	0.642820	0.381551	0.060*	0.52 (3)
C40	1.2119 (3)	0.79927 (16)	0.93629 (13)	0.0348 (4)	
H40A	1.324331	0.827142	0.955215	0.052*	0.83 (3)
H40B	1.222115	0.734965	0.890324	0.052*	0.83 (3)
H40C	1.153120	0.786971	0.984891	0.052*	0.83 (3)
H40D	1.142047	0.738910	0.931739	0.052*	0.17 (3)
H40E	1.244262	0.831087	0.996629	0.052*	0.17 (3)
H40F	1.313258	0.779081	0.902062	0.052*	0.17 (3)

### (90K) Atomic displacement parameters (Å^2^)

**Table:**

	*U*^11^	*U*^22^	*U*^33^	*U*^12^	*U*^13^	*U*^23^
N1	0.0278 (7)	0.0271 (8)	0.0333 (8)	−0.0018 (6)	−0.0007 (6)	0.0063 (6)
N2	0.0254 (7)	0.0303 (8)	0.0360 (8)	−0.0036 (6)	−0.0014 (6)	0.0082 (6)
C1	0.0230 (8)	0.0265 (9)	0.0304 (9)	−0.0058 (6)	0.0013 (6)	0.0108 (7)
C2	0.0207 (7)	0.0284 (9)	0.0334 (9)	−0.0018 (6)	−0.0004 (6)	0.0152 (7)
C3	0.0183 (7)	0.0246 (8)	0.0315 (8)	−0.0008 (6)	0.0031 (6)	0.0134 (7)
C4	0.0198 (7)	0.0236 (8)	0.0346 (9)	−0.0030 (6)	0.0015 (6)	0.0135 (7)
O1	0.0283 (6)	0.0260 (7)	0.0436 (7)	0.0049 (5)	0.0007 (5)	0.0146 (6)
N3	0.0227 (7)	0.0256 (7)	0.0308 (7)	−0.0018 (5)	0.0016 (5)	0.0101 (6)
C5	0.0181 (7)	0.0274 (9)	0.0320 (9)	−0.0006 (6)	0.0034 (6)	0.0142 (7)
O2	0.0253 (6)	0.0389 (7)	0.0318 (7)	0.0008 (5)	−0.0004 (5)	0.0159 (5)
C6	0.0165 (7)	0.0274 (9)	0.0312 (8)	−0.0024 (6)	0.0023 (6)	0.0151 (7)
C7	0.0195 (7)	0.0262 (8)	0.0344 (9)	0.0009 (6)	0.0041 (6)	0.0163 (7)
C8	0.0229 (8)	0.0238 (8)	0.0352 (9)	−0.0019 (6)	0.0049 (6)	0.0128 (7)
C9	0.0305 (9)	0.0247 (9)	0.0394 (10)	0.0004 (7)	0.0047 (7)	0.0106 (7)
C10	0.0279 (8)	0.0286 (9)	0.0344 (9)	−0.0001 (7)	0.0009 (7)	0.0084 (7)
C11	0.0268 (8)	0.0256 (9)	0.0255 (8)	−0.0007 (6)	0.0020 (6)	0.0037 (6)
C12	0.0239 (8)	0.0293 (9)	0.0254 (8)	−0.0001 (6)	0.0009 (6)	0.0049 (7)
C13	0.0288 (8)	0.0275 (9)	0.0235 (8)	−0.0030 (7)	0.0016 (6)	0.0035 (6)
C14	0.0341 (9)	0.0296 (9)	0.0243 (8)	−0.0056 (7)	0.0041 (7)	0.0026 (7)
O3	0.0305 (7)	0.0400 (8)	0.0391 (7)	−0.0077 (6)	0.0040 (6)	0.0084 (6)
N4	0.0371 (8)	0.0327 (9)	0.0271 (7)	−0.0076 (7)	0.0040 (6)	0.0067 (6)
C15	0.0395 (10)	0.0280 (9)	0.0227 (8)	−0.0016 (7)	0.0009 (7)	0.0046 (7)
O4	0.0467 (8)	0.0341 (8)	0.0350 (7)	−0.0019 (6)	−0.0030 (6)	0.0134 (6)
C16	0.0347 (9)	0.0252 (9)	0.0226 (8)	0.0000 (7)	0.0012 (7)	0.0034 (6)
C17	0.0297 (9)	0.0273 (9)	0.0271 (8)	0.0004 (7)	−0.0017 (7)	0.0042 (7)
C18	0.0279 (8)	0.0254 (8)	0.0265 (8)	−0.0029 (7)	0.0008 (6)	0.0029 (7)
C19	0.0310 (9)	0.0320 (10)	0.0344 (9)	−0.0025 (7)	0.0034 (7)	0.0063 (8)
C20	0.0478 (11)	0.0345 (10)	0.0322 (9)	−0.0102 (9)	0.0078 (8)	0.0088 (8)
N21	0.0277 (7)	0.0259 (8)	0.0418 (9)	−0.0003 (6)	0.0033 (6)	0.0048 (7)
N22	0.0292 (8)	0.0269 (8)	0.0440 (9)	0.0019 (6)	0.0013 (7)	0.0059 (7)
C21	0.0222 (8)	0.0251 (9)	0.0376 (9)	0.0032 (6)	−0.0012 (7)	0.0117 (7)
C22	0.0197 (7)	0.0266 (9)	0.0367 (9)	0.0001 (6)	0.0012 (6)	0.0144 (7)
C23	0.0201 (7)	0.0238 (8)	0.0354 (9)	0.0009 (6)	−0.0019 (6)	0.0153 (7)
C24	0.0212 (8)	0.0250 (9)	0.0371 (9)	0.0022 (6)	−0.0010 (6)	0.0134 (7)
O21	0.0293 (6)	0.0255 (7)	0.0467 (8)	−0.0037 (5)	0.0026 (5)	0.0127 (6)
N23	0.0211 (7)	0.0313 (8)	0.0345 (8)	0.0034 (6)	−0.0004 (6)	0.0102 (6)
C25	0.0216 (8)	0.0364 (10)	0.0334 (9)	0.0024 (7)	−0.0025 (7)	0.0173 (8)
O22	0.0248 (6)	0.0554 (9)	0.0354 (7)	0.0010 (6)	0.0026 (5)	0.0224 (6)
C26	0.0189 (7)	0.0307 (9)	0.0329 (9)	0.0004 (6)	−0.0020 (6)	0.0175 (7)
C27	0.0213 (8)	0.0324 (9)	0.0395 (10)	−0.0029 (7)	−0.0034 (7)	0.0223 (8)
C28	0.0256 (8)	0.0250 (9)	0.0418 (10)	−0.0019 (7)	−0.0068 (7)	0.0166 (8)
C29	0.0321 (9)	0.0254 (10)	0.0573 (12)	−0.0046 (7)	−0.0081 (8)	0.0175 (9)
C30	0.0330 (9)	0.0242 (9)	0.0417 (10)	−0.0046 (7)	0.0022 (8)	0.0097 (8)
C31	0.0300 (9)	0.0232 (9)	0.0282 (8)	−0.0034 (7)	−0.0006 (7)	0.0040 (7)
C32	0.0260 (8)	0.0282 (9)	0.0276 (8)	−0.0039 (7)	−0.0005 (6)	0.0060 (7)
C33	0.0286 (8)	0.0253 (9)	0.0242 (8)	−0.0031 (7)	−0.0012 (6)	0.0025 (6)
C34	0.0315 (9)	0.0285 (9)	0.0257 (8)	−0.0026 (7)	−0.0026 (7)	0.0037 (7)
O23	0.0276 (7)	0.0401 (8)	0.0412 (7)	0.0003 (5)	−0.0007 (5)	0.0134 (6)
N24	0.0334 (8)	0.0293 (8)	0.0285 (7)	0.0003 (6)	−0.0018 (6)	0.0072 (6)
C35	0.0360 (9)	0.0259 (9)	0.0259 (8)	−0.0035 (7)	0.0006 (7)	0.0037 (7)
O24	0.0448 (8)	0.0360 (8)	0.0390 (7)	−0.0032 (6)	0.0080 (6)	0.0158 (6)
C36	0.0322 (9)	0.0243 (9)	0.0238 (8)	−0.0047 (7)	0.0011 (6)	0.0031 (6)
C37	0.0280 (8)	0.0273 (9)	0.0288 (8)	−0.0041 (7)	0.0044 (7)	0.0032 (7)
C38	0.0270 (8)	0.0240 (9)	0.0287 (8)	−0.0017 (7)	0.0014 (6)	0.0007 (7)
C39	0.0320 (10)	0.0412 (12)	0.0408 (11)	0.0072 (8)	−0.0033 (8)	0.0027 (9)
C40	0.0406 (10)	0.0309 (10)	0.0337 (9)	0.0025 (8)	−0.0052 (8)	0.0111 (8)

### (90K) Geometric parameters (Å, º)

**Table:**

N1—N2	1.246 (2)	N21—N22	1.255 (3)
N1—C18	1.452 (2)	N21—C38	1.449 (2)
N2—C1	1.456 (2)	N22—C21	1.452 (2)
C1—C2	1.391 (3)	C21—C22	1.392 (3)
C1—C8	1.405 (3)	C21—C28	1.407 (2)
C2—C3	1.387 (2)	C22—C23	1.377 (3)
C2—H2	0.9500	C22—H22	0.9500
C3—C6	1.389 (2)	C23—C26	1.388 (2)
C3—C4	1.488 (2)	C23—C24	1.492 (2)
C4—O1	1.212 (2)	C24—O21	1.215 (2)
C4—N3	1.392 (2)	C24—N23	1.382 (2)
N3—C5	1.390 (2)	N23—C25	1.399 (2)
N3—C19	1.455 (2)	N23—C39	1.451 (3)
C5—O2	1.217 (2)	C25—O22	1.207 (2)
C5—C6	1.489 (2)	C25—C26	1.487 (3)
C6—C7	1.379 (3)	C26—C27	1.383 (3)
C7—C8	1.400 (3)	C27—C28	1.392 (3)
C7—H7	0.9500	C27—H27	0.9500
C8—C9	1.506 (3)	C28—C29	1.504 (3)
C9—C10	1.543 (2)	C29—C30	1.544 (3)
C9—H9A	0.9900	C29—H29A	0.9900
C9—H9B	0.9900	C29—H29B	0.9900
C10—C11	1.514 (3)	C30—C31	1.507 (3)
C10—H10A	0.9900	C30—H30A	0.9900
C10—H10B	0.9900	C30—H30B	0.9900
C11—C12	1.404 (2)	C31—C38	1.403 (2)
C11—C18	1.406 (3)	C31—C32	1.406 (3)
C12—C13	1.381 (3)	C32—C33	1.378 (3)
C12—H12	0.9500	C32—H32	0.9500
C13—C16	1.387 (3)	C33—C36	1.392 (2)
C13—C14	1.490 (2)	C33—C34	1.487 (3)
C14—O3	1.216 (2)	C34—O23	1.217 (2)
C14—N4	1.393 (3)	C34—N24	1.393 (2)
N4—C15	1.397 (3)	N24—C35	1.397 (3)
N4—C20	1.456 (2)	N24—C40	1.460 (3)
C15—O4	1.205 (2)	C35—O24	1.208 (2)
C15—C16	1.488 (3)	C35—C36	1.488 (3)
C16—C17	1.381 (3)	C36—C37	1.373 (3)
C17—C18	1.399 (3)	C37—C38	1.395 (3)
C17—H17	0.9500	C37—H37	0.9500
C19—H19A	0.9800	C39—H39A	0.9800
C19—H19B	0.9800	C39—H39B	0.9800
C19—H19C	0.9800	C39—H39C	0.9800
C19—H19D	0.9800	C39—H39D	0.9800
C19—H19E	0.9800	C39—H39E	0.9800
C19—H19F	0.9800	C39—H39F	0.9800
C20—H20A	0.9800	C40—H40A	0.9800
C20—H20B	0.9800	C40—H40B	0.9800
C20—H20C	0.9800	C40—H40C	0.9800
C20—H20D	0.9800	C40—H40D	0.9800
C20—H20E	0.9800	C40—H40E	0.9800
C20—H20F	0.9800	C40—H40F	0.9800
			
N2—N1—C18	119.17 (15)	N22—N21—C38	118.80 (16)
N1—N2—C1	120.42 (14)	N21—N22—C21	119.77 (16)
C2—C1—C8	123.02 (16)	C22—C21—C28	122.88 (17)
C2—C1—N2	118.01 (16)	C22—C21—N22	117.98 (16)
C8—C1—N2	118.36 (16)	C28—C21—N22	118.80 (17)
C3—C2—C1	116.58 (16)	C23—C22—C21	116.70 (16)
C3—C2—H2	121.7	C23—C22—H22	121.6
C1—C2—H2	121.7	C21—C22—H22	121.6
C2—C3—C6	121.18 (17)	C22—C23—C26	121.48 (17)
C2—C3—C4	130.69 (16)	C22—C23—C24	130.62 (15)
C6—C3—C4	108.10 (15)	C26—C23—C24	107.88 (16)
O1—C4—N3	125.28 (17)	O21—C24—N23	125.14 (17)
O1—C4—C3	128.95 (17)	O21—C24—C23	128.60 (17)
N3—C4—C3	105.77 (14)	N23—C24—C23	106.25 (14)
C5—N3—C4	112.23 (15)	C24—N23—C25	111.89 (15)
C5—N3—C19	123.10 (16)	C24—N23—C39	123.79 (16)
C4—N3—C19	123.93 (16)	C25—N23—C39	123.91 (16)
O2—C5—N3	124.94 (17)	O22—C25—N23	125.03 (19)
O2—C5—C6	129.18 (17)	O22—C25—C26	128.97 (18)
N3—C5—C6	105.87 (14)	N23—C25—C26	105.99 (15)
C7—C6—C3	122.15 (16)	C27—C26—C23	121.64 (17)
C7—C6—C5	129.92 (16)	C27—C26—C25	130.42 (16)
C3—C6—C5	107.92 (15)	C23—C26—C25	107.94 (16)
C6—C7—C8	118.10 (16)	C26—C27—C28	118.51 (16)
C6—C7—H7	120.9	C26—C27—H27	120.7
C8—C7—H7	120.9	C28—C27—H27	120.7
C7—C8—C1	118.93 (17)	C27—C28—C21	118.73 (17)
C7—C8—C9	122.41 (17)	C27—C28—C29	121.54 (17)
C1—C8—C9	118.65 (17)	C21—C28—C29	119.73 (18)
C8—C9—C10	113.34 (15)	C28—C29—C30	114.67 (16)
C8—C9—H9A	108.9	C28—C29—H29A	108.6
C10—C9—H9A	108.9	C30—C29—H29A	108.6
C8—C9—H9B	108.9	C28—C29—H29B	108.6
C10—C9—H9B	108.9	C30—C29—H29B	108.6
H9A—C9—H9B	107.7	H29A—C29—H29B	107.6
C11—C10—C9	118.20 (16)	C31—C30—C29	117.71 (15)
C11—C10—H10A	107.8	C31—C30—H30A	107.9
C9—C10—H10A	107.8	C29—C30—H30A	107.9
C11—C10—H10B	107.8	C31—C30—H30B	107.9
C9—C10—H10B	107.8	C29—C30—H30B	107.9
H10A—C10—H10B	107.1	H30A—C30—H30B	107.2
C12—C11—C18	118.15 (16)	C38—C31—C32	118.13 (17)
C12—C11—C10	118.09 (16)	C38—C31—C30	121.45 (17)
C18—C11—C10	123.54 (15)	C32—C31—C30	120.27 (16)
C13—C12—C11	118.56 (17)	C33—C32—C31	119.03 (16)
C13—C12—H12	120.7	C33—C32—H32	120.5
C11—C12—H12	120.7	C31—C32—H32	120.5
C12—C13—C16	121.96 (16)	C32—C33—C36	121.03 (18)
C12—C13—C14	130.29 (17)	C32—C33—C34	131.01 (16)
C16—C13—C14	107.66 (16)	C36—C33—C34	107.88 (16)
O3—C14—N4	125.86 (18)	O23—C34—N24	125.09 (19)
O3—C14—C13	128.46 (19)	O23—C34—C33	128.66 (19)
N4—C14—C13	105.69 (16)	N24—C34—C33	106.25 (15)
C14—N4—C15	112.75 (16)	C34—N24—C35	111.81 (16)
C14—N4—C20	124.15 (17)	C34—N24—C40	124.40 (16)
C15—N4—C20	123.09 (18)	C35—N24—C40	123.79 (17)
O4—C15—N4	126.50 (18)	O24—C35—N24	125.53 (19)
O4—C15—C16	128.53 (18)	O24—C35—C36	128.47 (18)
N4—C15—C16	104.91 (17)	N24—C35—C36	105.97 (15)
C17—C16—C13	121.37 (17)	C37—C36—C33	121.82 (17)
C17—C16—C15	129.62 (18)	C37—C36—C35	130.10 (16)
C13—C16—C15	108.95 (16)	C33—C36—C35	108.03 (16)
C16—C17—C18	116.52 (17)	C36—C37—C38	116.85 (16)
C16—C17—H17	121.7	C36—C37—H37	121.6
C18—C17—H17	121.7	C38—C37—H37	121.6
C17—C18—C11	123.28 (16)	C37—C38—C31	122.92 (17)
C17—C18—N1	113.43 (16)	C37—C38—N21	116.45 (16)
C11—C18—N1	123.04 (16)	C31—C38—N21	120.41 (17)
N3—C19—H19A	109.5	N23—C39—H39A	109.5
N3—C19—H19B	109.5	N23—C39—H39B	109.5
H19A—C19—H19B	109.5	H39A—C39—H39B	109.5
N3—C19—H19C	109.5	N23—C39—H39C	109.5
H19A—C19—H19C	109.5	H39A—C39—H39C	109.5
H19B—C19—H19C	109.5	H39B—C39—H39C	109.5
N3—C19—H19D	109.5	N23—C39—H39D	109.5
H19A—C19—H19D	141.1	H39A—C39—H39D	141.1
H19B—C19—H19D	56.3	H39B—C39—H39D	56.3
H19C—C19—H19D	56.3	H39C—C39—H39D	56.3
N3—C19—H19E	109.5	N23—C39—H39E	109.5
H19A—C19—H19E	56.3	H39A—C39—H39E	56.3
H19B—C19—H19E	141.1	H39B—C39—H39E	141.1
H19C—C19—H19E	56.3	H39C—C39—H39E	56.3
H19D—C19—H19E	109.5	H39D—C39—H39E	109.5
N3—C19—H19F	109.5	N23—C39—H39F	109.5
H19A—C19—H19F	56.3	H39A—C39—H39F	56.3
H19B—C19—H19F	56.3	H39B—C39—H39F	56.3
H19C—C19—H19F	141.1	H39C—C39—H39F	141.1
H19D—C19—H19F	109.5	H39D—C39—H39F	109.5
H19E—C19—H19F	109.5	H39E—C39—H39F	109.5
N4—C20—H20A	109.5	N24—C40—H40A	109.5
N4—C20—H20B	109.5	N24—C40—H40B	109.5
H20A—C20—H20B	109.5	H40A—C40—H40B	109.5
N4—C20—H20C	109.5	N24—C40—H40C	109.5
H20A—C20—H20C	109.5	H40A—C40—H40C	109.5
H20B—C20—H20C	109.5	H40B—C40—H40C	109.5
N4—C20—H20D	109.5	N24—C40—H40D	109.5
H20A—C20—H20D	141.1	H40A—C40—H40D	141.1
H20B—C20—H20D	56.3	H40B—C40—H40D	56.3
H20C—C20—H20D	56.3	H40C—C40—H40D	56.3
N4—C20—H20E	109.5	N24—C40—H40E	109.5
H20A—C20—H20E	56.3	H40A—C40—H40E	56.3
H20B—C20—H20E	141.1	H40B—C40—H40E	141.1
H20C—C20—H20E	56.3	H40C—C40—H40E	56.3
H20D—C20—H20E	109.5	H40D—C40—H40E	109.5
N4—C20—H20F	109.5	N24—C40—H40F	109.5
H20A—C20—H20F	56.3	H40A—C40—H40F	56.3
H20B—C20—H20F	56.3	H40B—C40—H40F	56.3
H20C—C20—H20F	141.1	H40C—C40—H40F	141.1
H20D—C20—H20F	109.5	H40D—C40—H40F	109.5
H20E—C20—H20F	109.5	H40E—C40—H40F	109.5
			
C18—N1—N2—C1	3.1 (3)	C38—N21—N22—C21	−0.4 (3)
N1—N2—C1—C2	−108.0 (2)	N21—N22—C21—C22	104.5 (2)
N1—N2—C1—C8	80.7 (2)	N21—N22—C21—C28	−82.0 (2)
C8—C1—C2—C3	0.3 (2)	C28—C21—C22—C23	0.0 (3)
N2—C1—C2—C3	−170.52 (14)	N22—C21—C22—C23	173.23 (16)
C1—C2—C3—C6	1.5 (2)	C21—C22—C23—C26	−2.0 (3)
C1—C2—C3—C4	178.86 (15)	C21—C22—C23—C24	179.93 (17)
C2—C3—C4—O1	5.6 (3)	C22—C23—C24—O21	−5.1 (3)
C6—C3—C4—O1	−176.78 (16)	C26—C23—C24—O21	176.64 (18)
C2—C3—C4—N3	−174.53 (16)	C22—C23—C24—N23	175.73 (17)
C6—C3—C4—N3	3.10 (17)	C26—C23—C24—N23	−2.54 (19)
O1—C4—N3—C5	176.38 (15)	O21—C24—N23—C25	−176.75 (17)
C3—C4—N3—C5	−3.50 (17)	C23—C24—N23—C25	2.46 (19)
O1—C4—N3—C19	6.0 (3)	O21—C24—N23—C39	−3.9 (3)
C3—C4—N3—C19	−173.93 (14)	C23—C24—N23—C39	175.32 (16)
C4—N3—C5—O2	−178.44 (15)	C24—N23—C25—O22	179.65 (17)
C19—N3—C5—O2	−7.9 (2)	C39—N23—C25—O22	6.8 (3)
C4—N3—C5—C6	2.54 (17)	C24—N23—C25—C26	−1.46 (19)
C19—N3—C5—C6	173.06 (14)	C39—N23—C25—C26	−174.31 (17)
C2—C3—C6—C7	−2.2 (2)	C22—C23—C26—C27	2.5 (3)
C4—C3—C6—C7	179.94 (14)	C24—C23—C26—C27	−179.01 (15)
C2—C3—C6—C5	176.28 (14)	C22—C23—C26—C25	−176.78 (16)
C4—C3—C6—C5	−1.62 (17)	C24—C23—C26—C25	1.68 (19)
O2—C5—C6—C7	−1.1 (3)	O22—C25—C26—C27	−0.6 (3)
N3—C5—C6—C7	177.83 (15)	N23—C25—C26—C27	−179.46 (17)
O2—C5—C6—C3	−179.41 (16)	O22—C25—C26—C23	178.60 (18)
N3—C5—C6—C3	−0.45 (17)	N23—C25—C26—C23	−0.23 (19)
C3—C6—C7—C8	0.9 (2)	C23—C26—C27—C28	−0.9 (3)
C5—C6—C7—C8	−177.17 (15)	C25—C26—C27—C28	178.22 (17)
C6—C7—C8—C1	0.9 (2)	C26—C27—C28—C21	−1.0 (3)
C6—C7—C8—C9	−178.32 (15)	C26—C27—C28—C29	178.33 (16)
C2—C1—C8—C7	−1.5 (2)	C22—C21—C28—C27	1.5 (3)
N2—C1—C8—C7	169.28 (15)	N22—C21—C28—C27	−171.66 (16)
C2—C1—C8—C9	177.72 (15)	C22—C21—C28—C29	−177.86 (17)
N2—C1—C8—C9	−11.5 (2)	N22—C21—C28—C29	9.0 (3)
C7—C8—C9—C10	103.58 (19)	C27—C28—C29—C30	−111.8 (2)
C1—C8—C9—C10	−75.6 (2)	C21—C28—C29—C30	67.5 (2)
C8—C9—C10—C11	24.9 (2)	C28—C29—C30—C31	−7.7 (3)
C9—C10—C11—C12	−132.48 (17)	C29—C30—C31—C38	−66.5 (2)
C9—C10—C11—C18	52.9 (2)	C29—C30—C31—C32	118.0 (2)
C18—C11—C12—C13	2.6 (2)	C38—C31—C32—C33	−3.6 (3)
C10—C11—C12—C13	−172.29 (16)	C30—C31—C32—C33	172.12 (16)
C11—C12—C13—C16	0.9 (3)	C31—C32—C33—C36	−0.3 (3)
C11—C12—C13—C14	176.91 (17)	C31—C32—C33—C34	−176.74 (17)
C12—C13—C14—O3	5.3 (3)	C32—C33—C34—O23	−5.6 (3)
C16—C13—C14—O3	−178.23 (19)	C36—C33—C34—O23	177.63 (19)
C12—C13—C14—N4	−175.21 (18)	C32—C33—C34—N24	175.08 (18)
C16—C13—C14—N4	1.23 (19)	C36—C33—C34—N24	−1.68 (19)
O3—C14—N4—C15	179.75 (18)	O23—C34—N24—C35	−179.27 (18)
C13—C14—N4—C15	0.3 (2)	C33—C34—N24—C35	0.1 (2)
O3—C14—N4—C20	−0.9 (3)	O23—C34—N24—C40	1.3 (3)
C13—C14—N4—C20	179.58 (16)	C33—C34—N24—C40	−179.33 (16)
C14—N4—C15—O4	175.77 (18)	C34—N24—C35—O24	−176.70 (18)
C20—N4—C15—O4	−3.5 (3)	C40—N24—C35—O24	2.7 (3)
C14—N4—C15—C16	−1.5 (2)	C34—N24—C35—C36	1.4 (2)
C20—N4—C15—C16	179.14 (16)	C40—N24—C35—C36	−179.15 (16)
C12—C13—C16—C17	−2.8 (3)	C32—C33—C36—C37	2.9 (3)
C14—C13—C16—C17	−179.63 (16)	C34—C33—C36—C37	−179.96 (16)
C12—C13—C16—C15	174.62 (16)	C32—C33—C36—C35	−174.60 (15)
C14—C13—C16—C15	−2.2 (2)	C34—C33—C36—C35	2.54 (19)
O4—C15—C16—C17	2.2 (3)	O24—C35—C36—C37	−1.6 (3)
N4—C15—C16—C17	179.48 (18)	N24—C35—C36—C37	−179.70 (18)
O4—C15—C16—C13	−174.94 (19)	O24—C35—C36—C33	175.58 (19)
N4—C15—C16—C13	2.31 (19)	N24—C35—C36—C33	−2.48 (19)
C13—C16—C17—C18	1.0 (3)	C33—C36—C37—C38	−1.3 (3)
C15—C16—C17—C18	−175.87 (17)	C35—C36—C37—C38	175.60 (17)
C16—C17—C18—C11	2.7 (3)	C36—C37—C38—C31	−2.9 (3)
C16—C17—C18—N1	177.19 (15)	C36—C37—C38—N21	−177.46 (15)
C12—C11—C18—C17	−4.6 (3)	C32—C31—C38—C37	5.3 (3)
C10—C11—C18—C17	170.06 (17)	C30—C31—C38—C37	−170.33 (17)
C12—C11—C18—N1	−178.51 (16)	C32—C31—C38—N21	179.70 (16)
C10—C11—C18—N1	−3.9 (3)	C30—C31—C38—N21	4.1 (3)
N2—N1—C18—C17	114.4 (2)	N22—N21—C38—C37	−113.5 (2)
N2—N1—C18—C11	−71.1 (2)	N22—N21—C38—C31	71.8 (2)

## References

[bb1] Brandenburg, K. (1999). *DIAMOND*. Crystal Impact GbR, Bonn, Germany.

[bb2] Bruno, I. J., Cole, J. C., Edgington, P. R., Kessler, M., Macrae, C. F., McCabe, P., Pearson, J. & Taylor, R. (2002). *Acta Cryst.* B**58**, 389–397. 10.1107/s0108768102003324 12037360

[bb3] Burk, M. H., Langbehn, D., Hernández Rodríguez, G., Reichstein, W., Drewes, J., Schröder, S., Rehders, S., Strunskus, T., Herges, R. & Faupel, F. (2021). *ACS Appl. Polym. Mater.* **3**, 1445–1456.

[bb4] Businski, A., Ta, T. C., Unterriker, L., Gindullis, N., von Glasenapp, J. S., Näther, C. & Herges, R. (2025). *Chem. Eur. J.* A**71**, 3–8. 10.1002/chem.202500435 PMC12099182 40090898

[bb5] Cabré, G., Garrido-Charles, A., González-Lafont, À., Moormann, W., Langbehn, D., Egea, D., Lluch, J. M., Herges, R., Alibés, R., Busqué, F., Gorostiza, P. & Hernando, J. (2019). *Org. Lett.* **21**, 3780–3784. 10.1021/acs.orglett.9b01222 31070376

[bb6] Corrado, F., Bruno, U., Prato, M., Carella, A., Criscuolo, V., Massaro, A., Pavone, M., Muñoz-García, A. B., Forti, S., Coletti, C., Bettucci, O. & Santoro, F. (2023). *Nat. Commun.* **14**, 6760–6760. 10.1038/s41467-023-41083-2 PMC10622443 37919279

[bb7] Deng, J., Wu, X., Guo, G., Zhao, X. & Yu, Z. (2020). *Org. Biomol. Chem.* **18**, 5602–5607. 10.1039/d0ob01027h 32647842

[bb8] Duval, H. (1910). *Bull. Soc. Chim. Fr.* **7**, 727–732.

[bb9] Ewert, J., Heintze, L., Jordà-Redondo, M., von Glasenapp, J. S., Nonell, S., Bucher, G., Peifer, C. & Herges, R. (2022). *J. Am. Chem. Soc.* **144**, 15059–15071. 10.1021/jacs.2c03649 35952371

[bb10] Groom, C. R., Bruno, I. J., Lightfoot, M. P. & Ward, S. C. (2016). *Acta Cryst*. B**72**, 171–179. 10.1107/S2052520616003954 PMC4822653 27048719

[bb11] Joshi, D. K., Mitchell, M. J., Bruce, D., Lough, A. J. & Yan, H. (2012). *Tetrahedron* **68**, 8670–8676.

[bb12] Jun, M., Joshi, D. K., Yalagala, R. S., Vanloon, J., Simionescu, R., Lough, A. J., Gordon, H. L. & Yan, H. (2018). *Chem. Sel.* **3**, 2697–2701.

[bb13] Krämer, R., Nöthling, N., Lehmann, C. W., Mohr, F. & Tausch, M. W. (2018). *ChemPhotoChem* **2**, 6–11.

[bb14] Lancia, F., Ryabchun, A. & Katsonis, N. (2019). *Nat. Rev. Chem.* **3**, 536–551. 10.1038/s41570-022-00392-8 37117430

[bb15] Li, S., Bamberg, K., Lu, Y., Sönnichsen, F. D. & Staubitz, A. (2023). *Polymers* **15**, 1306. 10.3390/polym15051306 PMC10007058 36904547

[bb16] Liu, Y., Li, F., Li, D., Dong, W. & Jin, B. (2023). *J. Electroanal. Chem.* **944**, 117644.

[bb17] López-Cano, M., Scortichini, M., Tosh, D. K., Salmaso, V., Ko, T., Salort, G., Filgaira, I., Soler, C., Trauner, D., Hernando, J., Jacobson, K. A. & Ciruela, F. (2024). *J. Am. Chem. Soc.* **147**, 874–879. 10.1021/jacs.4c13558 PMC11726555 39680577

[bb18] Martino, S., Mauro, F. & Netti, P. A. (2020). *Riv. Nuovo Cim.* **43**, 599–629.

[bb19] Moormann, W., Tellkamp, T., Stadler, E., Röhricht, F., Näther, C., Puttreddy, R., Rissanen, K., Gescheidt, G. & Herges, R. (2020). *Angew. Chem. Int. Ed.* 2020 **59**, 15081–15086. 10.1002/anie.202005361 PMC7496762 32348617

[bb20] Mukherjee, A., Seyfried, M. D. & Ravoo, B. J. (2023). *Angew. Chem. Int. Ed.* **62**, e202304437. 10.1002/anie.202304437 37212536

[bb21] Paudler, W. W. & Zeiler, A. G. (1969). *J. Org. Chem.* **34**, 3237–3239.

[bb22] Perlllmutter, H. D. (1990). *Adv. Heterocycl. Chem.* **50**, 1–83.

[bb23] Rigaku OD (2023). *CrysAlis PRO*. Rigaku Oxford Diffraction, Yarnton, England.

[bb24] Sheldrick, G. M. (2008). *Acta Cryst.* A**64**, 112–122. 10.1107/S0108767307043930 18156677

[bb25] Sheldrick, G. M. (2015*a*). *Acta Cryst.* A**71**, 3–8.

[bb26] Sheldrick, G. M. (2015*b*). *Acta Cryst.* C**71**, 3–8.

[bb27] Siewertsen, R., Neumann, H., Buchheim-Stehn, B., Herges, R., Näther, C., Renth, F. & Temps, F. (2009). *J. Am. Chem. Soc.* **131**, 15594–15595. 10.1021/ja906547d 19827776

[bb28] Siewertsen, R., Schönborn, J. B., Hartke, B., Renth, F. & Temps, F. (2011). *Phys. Chem. Chem. Phys.* **13**, 1054–1063. 10.1039/c0cp01148g 21072405

[bb29] Westrip, S. P. (2010). *J. Appl. Cryst.* **43**, 920–925.

[bb30] Zhu, Q. (2020). *CSD Communication* (CCDC 1902659). CCDC, Cambridge, England.

[bb31] Zhu, Q., Wang, S. & Chen, P. (2019). *Org. Lett.* **21**, 4025–4029. 10.1021/acs.orglett.9b01215 31084009

